# The plausibility of claimed induced seismicity

**DOI:** 10.1038/s41598-024-81632-3

**Published:** 2024-12-28

**Authors:** Max Wilkinson, Najwa Mhana, Miles P. Wilson, Gillian R. Foulger, Timur Tezel, Jon G. Gluyas

**Affiliations:** 1 Foulger Consulting Ltd., Durham, UK; 2 Damascus University, Damascus, Syria; 3 Department of Earth Sciences, Science Labs, Durham University, Durham, UK; 4 Department of Earth Sciences, Science Labs, Sakarya University, Sakarya, Türkiye; 5 Geoenergy Durham Ltd., Durham, UK; 6 School of Engineering, Newcastle University, Newcastle upon Tyne, UK

**Keywords:** Natural hazards, Solid Earth sciences

## Abstract

Claims of industrially induced seismicity vary from indisputable to unpersuasive and yet the veracity of industrial induction is vital for regulatory and operational practice. Assessment schemes have been developed in response to this need. We report here an initial assessment of the reliability of all globally known cases of proposed human-induced earthquakes and invite specialists on particular cases to refine these results. 1235 cases were assessed, requiring over 1000 h of work. From the 881 cases for which scorable evidence is available, we class 87% as ‘Confidently Induced’, 10% as ‘Probably Induced’, 2% as ‘Equivocal’ and < 1% as ‘Confidently Natural’. The most seismogenic activities are fracking, research, geothermal, water reservoir impoundment, conventional oil and gas. Least seismogenic activities are construction, deep penetrating bombs, coal bed methane. 354 cases (29%) lack enough information to be assessable. Future work could include applying data mining techniques including natural language processing and AI to uncover new evidence. Future best practice for rapid assessment of cases would ideally involve an independent panel of scientists who rapidly apply a questionnaire scheme, reach consensus, and inform a response.

**Supplementary Information:**

The online version contains supplementary material available at 10.1038/s41598-024-81632-3.

## Main

Human-induced earthquakes are a global phenomenon caused by various activities^1–7^. Their negative effects vary from societal nuisance^8^ to major economic losses and human fatalities^9^. There are major challenges in predicting the occurrence and magnitude of induced earthquakes. It is, however, very likely that they will become more commonplace as human populations grow and Earth’s resources are increasingly exploited. To date, over 1200 scientifically proposed cases of induced seismicity have been documented globally^10^. The scientific evidence, and thus the reliability of claims for induced earthquakes, is highly variable in completeness and quality. Temporal trends and bias exist whereby interest in such earthquakes is linked to the proliferation of causative activities and their associated controversies. Global interest, often in terms of opposition, to fracking of shales and other low-permeability petroleum reservoirs expanded from around 2012 and increased public awareness of induced earthquakes^11^.

In the absence of universally applicable quantitative approaches to determine case reliability, questionnaire schemes have been developed for specific seismogenic activities^12^. These have evolved in complexity and applicability^13–15^. The most recent such scheme, “Evaluating Proposals of Induced Earthquakes (*E-PIE*)”, was developed to be universally applicable regardless of causative activity. *E-PIE* is objective, based entirely on the strength of claimed evidence, and de-emphasises the personal opinions of the assessor^16^. Such schemes are becoming critical tools for industries where operators and regulators recognise the potential for induced seismicity and need to act rapidly if it occurs.

In this study we provide an initial, comprehensive, standardised evidence assessment of all currently known, worldwide cases of induced seismicity. A single assessor independently applied the *E-PIE* scheme to 1235 proposed cases in the Human-Induced Earthquake Database (*HiQuake*). This publicly available database is the largest and most complete compilation of scientifically proposed human-induced earthquakes^17–19^. The assessment took over 1000 h of study. A preferred approach would have been to use an expert panel to assess all 1235 cases, but this would have required excessive resources. The application of a consistent scheme by a single assessor provides a uniform, preliminary set of results suitable for assessing induced seismicity through time and across different industries.

Compromise was required in applying a standardised assessment scheme to the broadest range of cases and the contentious nature of such work. The results should thus be viewed as initial and provisional. We invite feedback from experts on individual cases so these results, which we provide publicly in the *HiQuake* database, may be improved.

## Results

### Scoring distribution by question

**Fig. 1:**
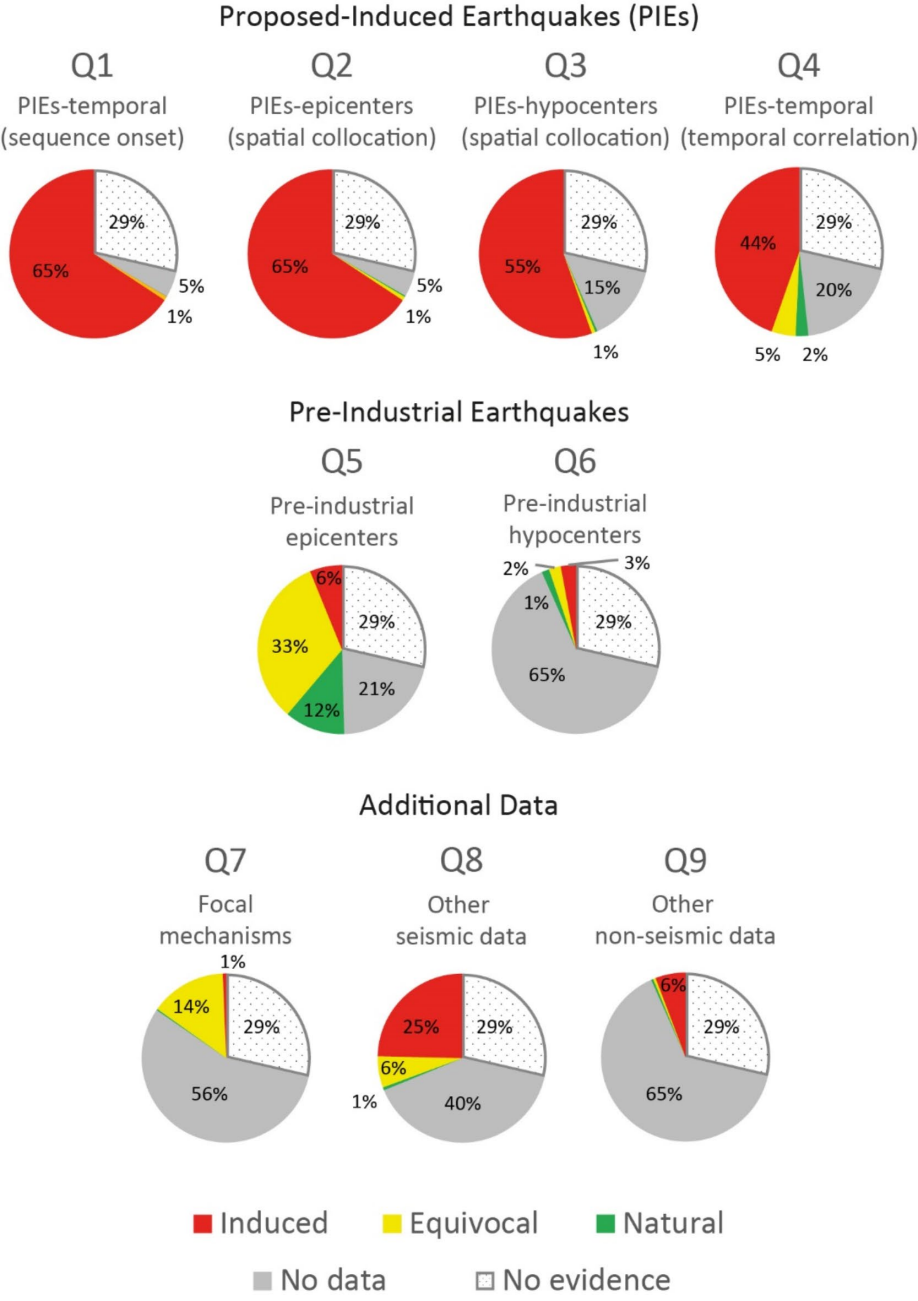
Distribution of scores for each of the nine *E-PIE* Questions for 1235 cases in *HiQuake* conducted by the single assessor. Dotted fields indicate ‘no evidence’ scores where the assessor was unable to answer any of the nine *E-PIE* Questions.

**Fig. 2:**
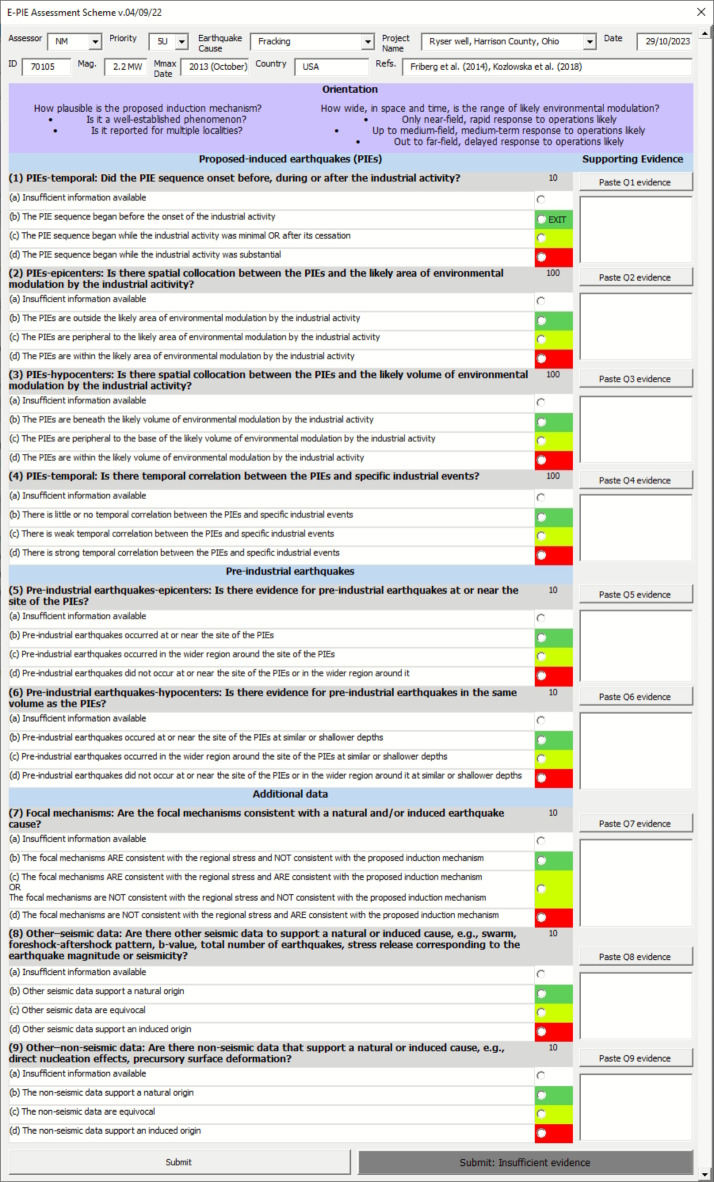
The interactive form used in the *E-PIE* assessment.

We analysed the distribution of assessor responses (Fig. 1) to each of the nine questions of the *E-PIE* scheme (Fig. 2). *E-PIE* comprises nine generalized questions, responses to which indicate the likelihood that the earthquakes were induced by human activity^16^. Of the 1235 cases studied, the assessor found there was insufficient evidence to score *any* question for 354 cases (29%). These were scored as ‘no evidence’ across all nine questions (Fig. 1, grey dotted area). Such cases are commonly presented in tables without supporting data, a common situation for water reservoir impoundment^3,20^, mining^21,22^, conventional oil and gas^5^ and nuclear explosions^23^. Questions 1–4, which refer to the temporal and spatial distributions of the seismicity in relation to the activity, were dominated by induced evidence (45–66%). Questions 6–9, which refer to auxiliary seismic parameters such as focal mechanisms, seismic swarm evolution and surface deformation, were most lacking of evidence (40–65%). Question 5, which relates to pre-industrial seismicity, had the greatest proportion of scores that were ‘equivocal’ (i.e. regional seismicity occurred before the activity, 33%) and ‘natural’ (i.e. local seismicity occurred before the activity, 12%).

### Cluster analysis

**Fig. 3:**
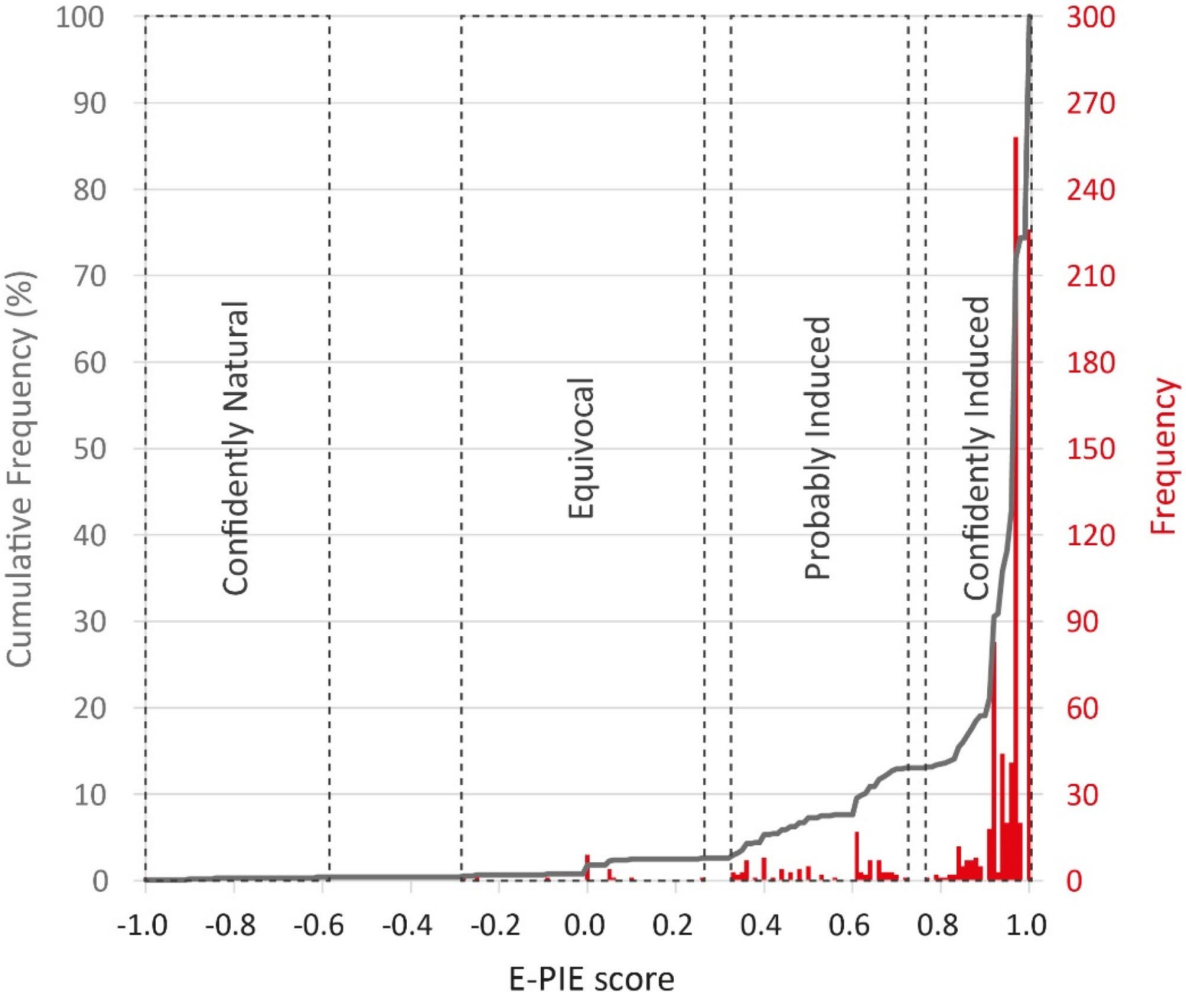
Visualisation of the four clusters overlain on frequency and cumulative frequency plots for the 881 cases in *HiQuake* which could be scored.

Summary scores for each case were calculated using an assessment tool developed to streamline the workflow (Online Methods: *Application of E-PIE scheme*). We found a continuum of results ranging from ‘confidently natural earthquakes’ (-1 score), through equivocal (0 score), to ‘confidently induced earthquakes’ (+ 1 score)^16^. For classification, we looked for potential clustering that might enable evidence-based division of the results into likely induced cases and likely natural cases (Online Methods: *Cluster analysis*). Excluding the ‘no evidence’ cases, we found a 4-cluster optimisation based on a Goodness of Variance Fit (GVF) of 0.93 to be the minimum possible number of clusters to represent the data (Figure S1a, triangular data point, table S2). We name these Cluster 1: Confidently Natural; Cluster 2: Equivocal; Cluster 3: Probably Induced; Cluster 4: Confidently Induced. Their bounds are shown in the frequency/cumulative-frequency plot of Fig. 3.

### Scoring distribution by process

**Fig. 4:**
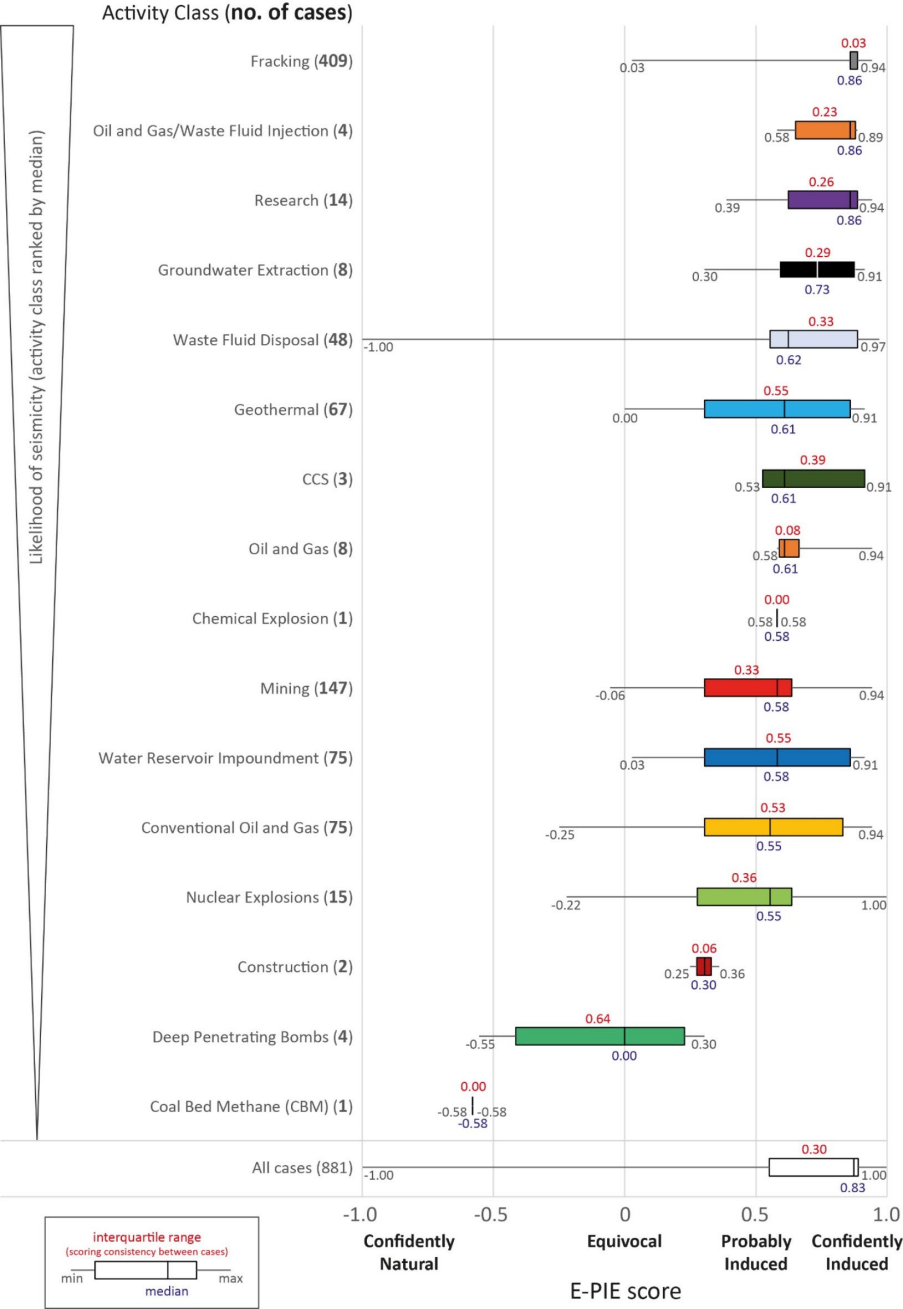
Number, *E-PIE* scores and summary statistics for cases within for each of the 16 activity classes in *HiQuake*. Classes are independent of one another and arranged by their median, which has not been normalised.

All *HiQuake* cases are linked to one of 16 activities^10^ (Table 1). We explored the *E-PIE* scores and statistics for each of these activities (Fig. 4; Online Methods: *Application of E-PIE scheme*). There was significant overlap in the range of *E-PIE* scores for cases within most activities. However, there were differences between activities. Figure 4 shows activities in order of reducing median *E-PIE* score. Cases in fracking, research, oil and gas/waste fluid injection, and groundwater extraction mostly lay in the Confidently Induced range with fracking being most numerous. Cases in waste fluid disposal, geothermal, and carbon capture and storage (CCS) activities generally spanned the range from Confidently Induced to Probably Induced. Cases in oil and gas, chemical explosions, mining, water reservoir impoundment, conventional oil and gas, and nuclear explosions activities were mostly within the Probably Induced range. Construction lies on the boundary between Probably Induced and Equivocal, whilst deep penetrating bombs was mostly within the Equivocal range. Coal bed methane (CBM) comprises a single case entirely within the natural range. Nuclear explosions contained the highest-scored case (the Cannikin test: 1.00). The lowest scored case is the Center, Texas, waste fluid disposal case where the earthquake sequence began before the industrial activity. In this situation the *E-PIE* assessment stopped at Question 1 because it triggered an exit criterion, resulting in the case being scored as natural (*E-PIE* score − 1.00).

**Table 1:** The 16 activity classes in *HiQuake* and their impact on the shallow crust.

Activity Class	Impact on shallow crust
CCS	Pore-pressure increase
Chemical explosion	Inelastic radial deformation to form explosion cavity, subsequent collapse
Coal Bed Methane (CBM)	Pore-pressure decrease (extraction)
Construction	Vertical loading and compaction, pore-pressure increase by fluid migration
Conventional Oil and Gas	Pore-pressure decrease
Deep penetrating bombs	Inelastic radial deformation to form explosion cavity, subsequent collapse
Fracking	Pore-pressure increase
Geothermal	Pore-pressure increase (injection), pore-pressure decrease (production), thermal changes
Groundwater extraction	Pore-pressure decrease (extraction)
Mining	Vertical unloading, pore-pressure decrease, cavity collapse
Nuclear explosions	Inelastic radial deformation to form explosion cavity, subsequent collapse
Oil and Gas	Pore-pressure increase (injection)
Oil and Gas/Waste fluid injection	Pore-pressure increase (injection)
Research	Pore-pressure increase (injection), pore-pressure cycling (stimulation)
Waste fluid disposal	Pore-pressure increase (injection)
Water reservoir impoundment	Vertical loading and compaction, pore-pressure increase by fluid migration

The activities chemical explosions, construction, and coal bed methane had small interquartile ranges due to small case numbers. Fracking was notable for its narrow interquartile range and large number of cases. Deep penetrating bombs, geothermal, water reservoir impoundment, and conventional oil and gas had the widest interquartile ranges.

### Logical conflicts

Logical conflicts within the *E-PIE* assessment occur when there are contradictory responses to individual Questions. They indicate inconsistent evidence or assessor error and therefore act to quality check the assessment. We validated the assessment of each case using conditional logic to identify logical conflicts (see Online Methods: *Logical conflicts*). Of the 1235 cases assessed, only one logical conflict was detected – the fracking case of Fox Creek SS14. The conflict existed between a ‘natural’ score for Question 7 and an ‘induced’ score for Question 4 (Fig. 2). Reassessment of the case changed the Question 7 score to ‘equivocal’, resolving the conflict.

## Discussion

### Scientifically proposed cases are mostly reasonable

Of the 881 cases in *HiQuake* presenting scorable evidence using *E-PIE*, 87% (766) fall within the category ‘Confidently Induced’, with a range of *E-PIE* scores of 0.77–1.00 (Fig. 3, Table S2). Most cases in *HiQuake* therefore represent reasonable proposals. The remaining lie in a broad spectrum with three discrete clusters (Fig. 3, Table S2): 10% (92) lie in the ‘Probably Induced’ category, 2% (19) in the ‘Equivocal’ category, and < 1% (4) in the ‘Confidently Natural’ category. ‘Confidently Induced’ cases were present for all activities except chemical explosions, construction, deep penetrating bombs, and coal bed methane (Fig. 4). The most seismogenic activities are fracking, research, geothermal (i.e. stimulation, production, injection and circulation), water reservoir impoundment, and conventional oil and gas.

### Key questions for determining induced or natural cause

The majority of evidence presented in favour of induced seismicity relates to *E-PIE* questions 1–4 (Figs. 1 and 2) which address the temporal and spatial nature of earthquakes relative to the industrial activity. These four questions are the most influential in determining whether the earthquakes are induced^16^. To reflect this importance in *E-PIE*, these questions utilise an exit criterion in question 1 and increased weighting in questions 2–4. Questions 5 and 6 examine whether the area was seismically active prior to the implicated activity (Fig. 2). If so, this weakens the case that earthquakes were induced (Fig. 1). These questions are least prominent in the evaluation of mining and water reservoir impoundment cases where pre-existing seismicity is commonly not considered or presented. Questions 7–9 provide the opportunity to add evidence from additional data (Fig. 2). These data may comprise analyses not yet developed, thus ensuring *E-PIE* remains applicable if new methods are developed. These questions also enable inclusion of diverse information from a range of activities. Well-documented activities including fracking, geothermal, groundwater extraction, nuclear explosions, oil and gas, research, waste fluid disposal, and water reservoir impoundment commonly provide evidence that can be input into these questions.

*E-PIE* scores vary widely within each activity class (Fig. 4). The median score and interquartile range for each activity distinguishes the least-seismogenic (construction, deep penetrating bombs and coal bed methane) from more-seismogenic activities. The single coal bed methane case is the only one scored as natural because of the earthquakes’ distance and the induced stress field being incompatible with the focal mechanism^24^. There is significant overlap in the results for the remaining activity classes such that the *E-PIE* score of any individual case is not a reliable indicator of the claimed activity. This implies that for induced seismicity in areas of mixed activity, the assessment results cannot be used to determine the causative activity.

The ranking of activities based on their median score (Fig. 4) provides an indication of the likelihood seismicity was induced when considering the evidence collated within *HiQuake* and the variable numbers of individual cases within each activity. The interquartile range for each activity provides insight into scoring consistency during this initial assessment. Considering median and interquartile range in this context, fracking (409 cases), oil and gas/waste fluid injection (4 cases), and research (14 cases) are scored as Confidently Induced with relatively narrow ranges due to consistently comprehensive documentation of evidence.

### Utilisation of E-PIE and other questionnaire schemes

Major advantages of questionnaire schemes such as *E-PIE* are their simplicity and rapid application. Whereas detailed scientific studies might provide more robust assessments, rarely are these possible within the hours to days required by operators or regulatory bodies during activities. This problem was highlighted by the 2022 M_W_ 5.2 Peace River earthquake, Alberta, Canada. The Alberta Energy Regulator initially stated the earthquake was natural^25^ but later conclusively demonstrated it was induced by wastewater disposal. The latter conclusion was also reached retrospectively by an expert panel applying both the Verdon^15^ and *E-PIE* schemes^26^.

Rapid assessment holds value in sometimes producing unexpected results that may prompt further examination and discussion. Best practice for rapid assessment of potentially induced earthquakes may comprise a readily available independent panel of scientists across regulation, industry, and academia. Panel members may apply the same questionnaire scheme and consensus may inform the course of action. A statistical analysis of the variation in questionnaire results between individual scientists and schemes, and the effect of averaging results, has been presented^16^. An independent panel could also debate historic cases of induced seismicity. Repeat assessments of cases by the same, or new panels, could be carried out as needed to consider new observations, data, and/or analytical methods.

### Cases lacking published evidence

A significant proportion (29%) of cases in *HiQuake* lack supporting evidence, despite being proposed in scientific literature, and thus could not be assessed using *E-PIE*. These cases are commonly listed in published tables of known induced seismicity and hence included in *HiQuake*. They are prevalent in the activities water reservoir impoundment^3,20^, mining^21,22^, conventional oil and gas^5^ and nuclear explosions^23^. Cases may be based on general opinion, or the supporting evidence may lie in un-referenced or unpublished material. We made every effort to include all material available during the assessment process. However, significant potential clearly exists to extend the results to these cases by applying data mining techniques such as natural language processing and artificial intelligence to uncover new or overlooked evidence in the vast literature that now exists.

## Online methods

### Application of the E-PIE scheme

The *E-PIE* questionnaire scheme^16^ (Fig. 2) was applied to the entire *HiQuake* database of proposed human-induced earthquakes. *HiQuake* contained 1235 cases as of 10th December 2021, the version used for this study. A single assessor with a Ph.D. in earthquake seismology worked for around 1000 h over a 20-month period to perform this task. To facilitate the assessment process and data management of the results, *E-PIE* was coded into an interactive form. Using this form, each of the nine questions in *E-PIE* must be answered with one of four responses: ‘no data’, ‘natural’, ‘equivocal’ or ‘induced’. Answers represent the evidence proposed in the scientific literature and not the assessor’s opinion of this evidence. In this way, the results are as objective as possible.

The scientific literature used to assess each case was illustrative, not comprehensive, due to the vast volume that exists for many cases. For publication-rich cases only prominent and commonly cited literature (e.g. publications in leading peer-reviewed scientific journals commonly cited throughout induced seismicity research) were included. In cases where no data were found to answer any *E-PIE* question, the case was labelled ‘insufficient evidence’ to distinguish it from cases where individual questions are scored ‘no data’. In the rare instances where proposed evidence for a question was contradictory, the assessor prioritised the proposed evidence relating to the most rigorous scientific process, including validation of results using additional methodologies. Where proposed evidence was contradictory but considered of equal credibility the assessor recorded a response of ‘equivocal’.

To visualise and interpret the collective results of the *E-PIE* assessments, a single quantifiable score was calculated for each case^16^ as follows. The following scores were applied to the response to each question: 0 for a (no data), -1 for b (natural), 0 for c (equivocal), and 1 for d (induced). The scores are then normalised by their *E-PIE* weightings for each question (Questions 1 and 5–9 are each weighted 1/36th and questions 2, 3 and 4 are each weighted 10/36th ). Summing produces an aggregate score analogous to the Induced Assessment Ratio (IAR)^15^. The weighted proportion of ‘no data’ responses is totalled and subtracted from 1 to produce a value of ‘coverage’ in the range of 0 to 1. The aggregate score is then multiplied by the coverage to produce the final *E-PIE* score in the range − 1 (natural), through 0 (equivocal) to + 1 (induced).

The results were interpreted by question and activity. For each of the 16 activities, summary statistics of the minimum, maximum, first quartile, third quartile and median value were calculated.

### Cluster analysis

Calculated *E-PIE* scores of the 1235 *HiQuake* cases yield a continuum from natural (-1), through equivocal (0), to induced (+ 1). Clustering exists within this continuum, providing an objective way to informally group and reference cases. Jenks Natural Break Optimisation^27^ was used to determine discrete clusters, whereby the variance within each cluster is minimised while the variance between clusters is maximised. The optimisation is expressed as the Goodness of Variance Fit (GVF), with values calculated for cluster sizes two through eight. Four clusters, with GVF of 0.93, were selected as the minimum number of clusters with sufficient GVF. This maintained most cluster break points in the six- and seven-cluster optimisations, while minimising inter-cluster spacing (Figure S1a, Table S2, Figure S5). Kernel density estimation (KDE)^28^ was conducted for the *HiQuake* population, which supports the four-cluster optimisation (Figure S1b).

We checked for underlying clustering inherent to the *E-PIE* scheme by producing a population of all possible permutations of *E-PIE* (Figure S3a) and by taking a random subset of the 1235 scores (Figure S3b). Comparable cluster analysis^27^ and KDE^28^ was conducted with the random subset (Figure S4a, S4b) and the results compared to those utilising the *HiQuake* population (Figure S5). Cluster break points within the random subset and *HiQuake* populations were found not to coincide and crucially the *HiQuake* clusters spanned break points from the random subset in multiple cases across all cluster optimisations (Figure S5). This demonstrates that clustering inherent to the *E-PIE* scheme did not significantly influence the *HiQuake* population and the resultant cluster analysis.

### Logical conflicts

The nine *E-PIE* questions were answered independently by the assessor. Each question is designed to be independent and not overlap logically with other questions since this would result in some data being counting twice (with the exception of question 1 when the response ‘natural’ is selected^16^). The combinations of plausible logical conflicts^16^ within *E-PIE* are shown in Table S6. ‘Natural’ responses to questions 1, 2, 3 or 7 (Fig. 2) imply a natural origin is required and ‘induced’ responses to questions 4 or 7 (Fig. 2) imply an induced origin is required. All other responses allow ambiguity in the final result and so either solution is permitted.

A conditional statement was used to search for logical conflicts within the assessor’s responses for each case. A logical conflict exists if the following conditional statement (written here in no specific code) is met:


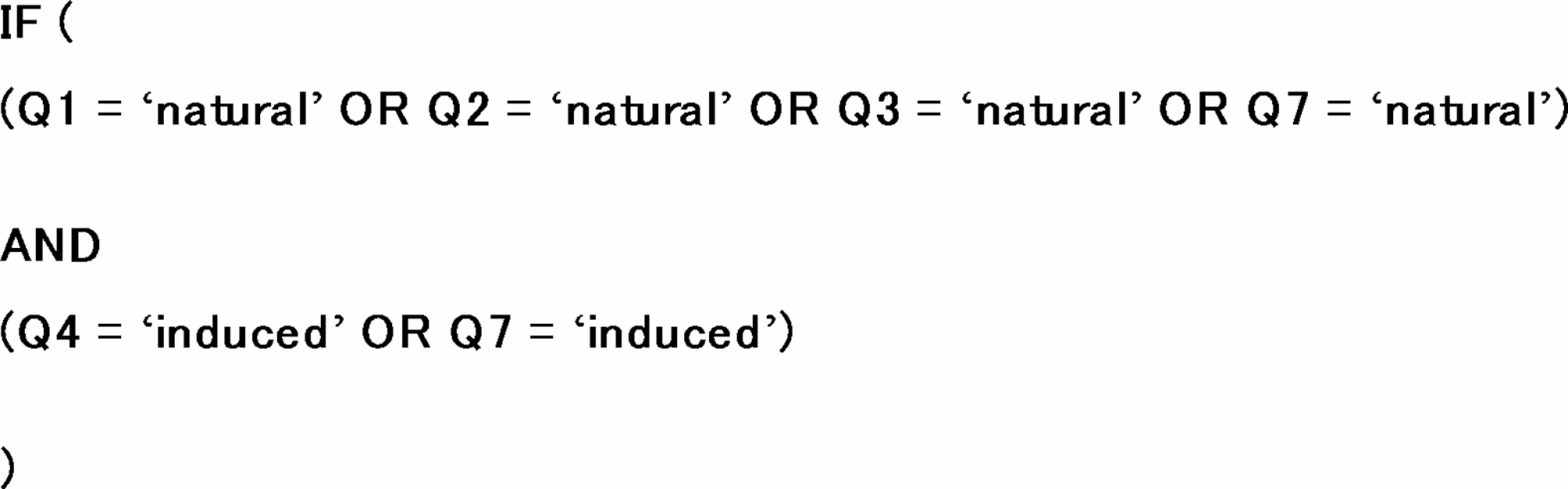


## Electronic supplementary material

Below is the link to the electronic supplementary material.

Supplementary Material 1

Supplementary Material 2

## Data Availability

The HiQuake database is freely available to download from www.inducedearthquakes.org. The E-PIE scores are available within the supplementary data and will be included online in a future update of HiQuake.
