# Supplementary material for: The plausibility of claimed induced seismicity
Source: Sci Rep. 2024 Dec 28;14:30846. doi: 10.1038/s41598-024-81632-3 (PMC11681127; doi:10.1038/s41598-024-81632-3)
Supplement: Supplementary file 2 — Supplementary Material 2 [file 41598_2024_81632_MOESM2_ESM.docx]

**Supplementary material**

**
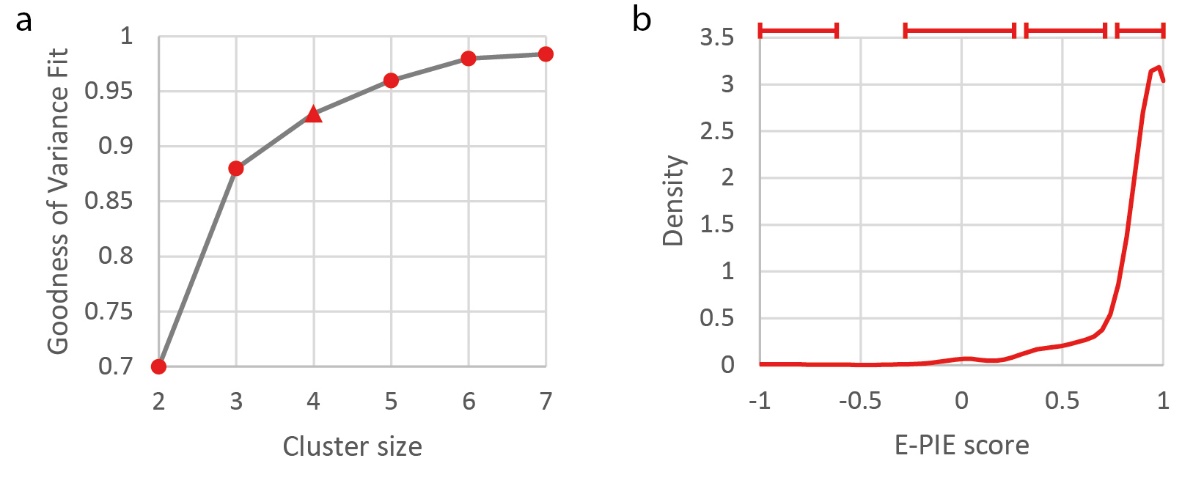
**

Figure S1: For the *HiQuake* cases scored in this work: (a) Goodness of Variance Fit using Jenks natural breaks^27^ for cluster sizes two to seven. The triangle data point for cluster size four represents the chosen optimisation. (b) Kernel density estimation^28^ for the population. Break points of the chosen four-cluster optimisation shown by the red bar at the top of the chart.

Table S2: Cluster boundaries and number of cases within each, of the chosen four-cluster optimisation using Jenks natural breaks^27^ for *HiQuake* cases in this work (Figure S1a, red triangle).

| **Cluster** | **Lower boundary** | **Upper boundary** | **Number of cases** |
| --- | --- | --- | --- |
| 1: Confidently Natural | -1.00 | -0.62 | 4 |
| 2: Equivocal | -0.28 | 0.26 | 19 |
| 3: Probably Induced | 0.32 | 0.71 | 92 |
| 4: Confidently Induced | 0.77 | 1.00 | 766 |


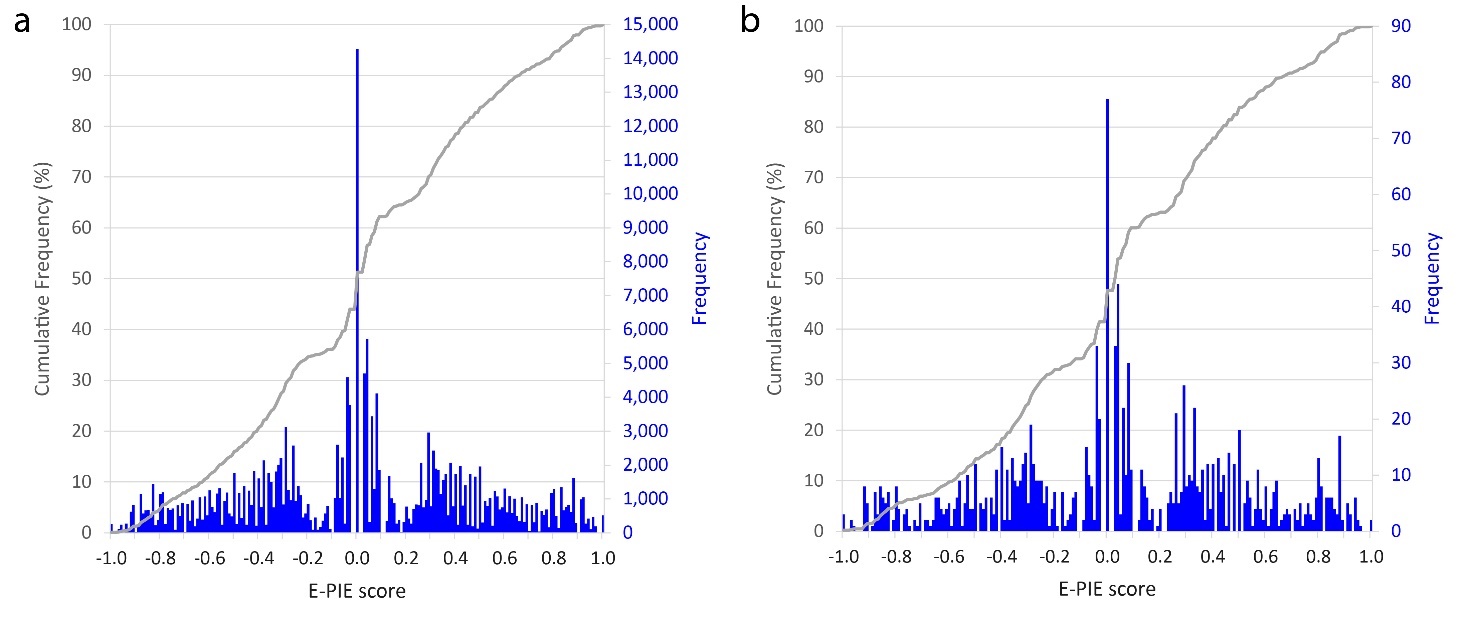


Figure S3: Frequency and cumulative frequency plots of (a) all possible permutations of *E-PIE* and (b) a random subset of 1,235 scores taken from the population shown in (a).


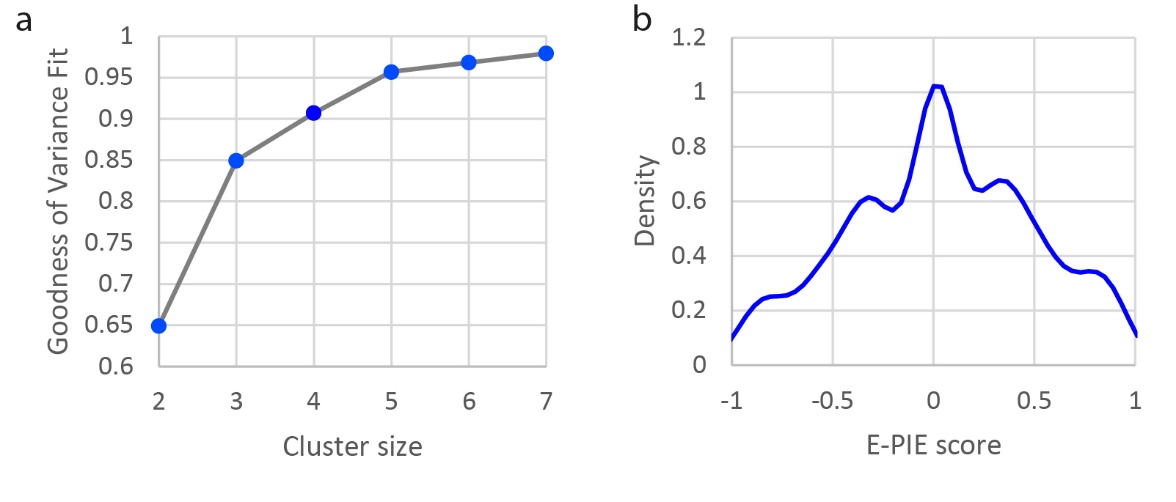


Figure S4: For the random subset dataset in S3(b), (a) Goodness of Variance Fit using Jenks natural breaks^27^ for cluster sizes two to seven, and (b) Kernel density estimation^28^ for the population.


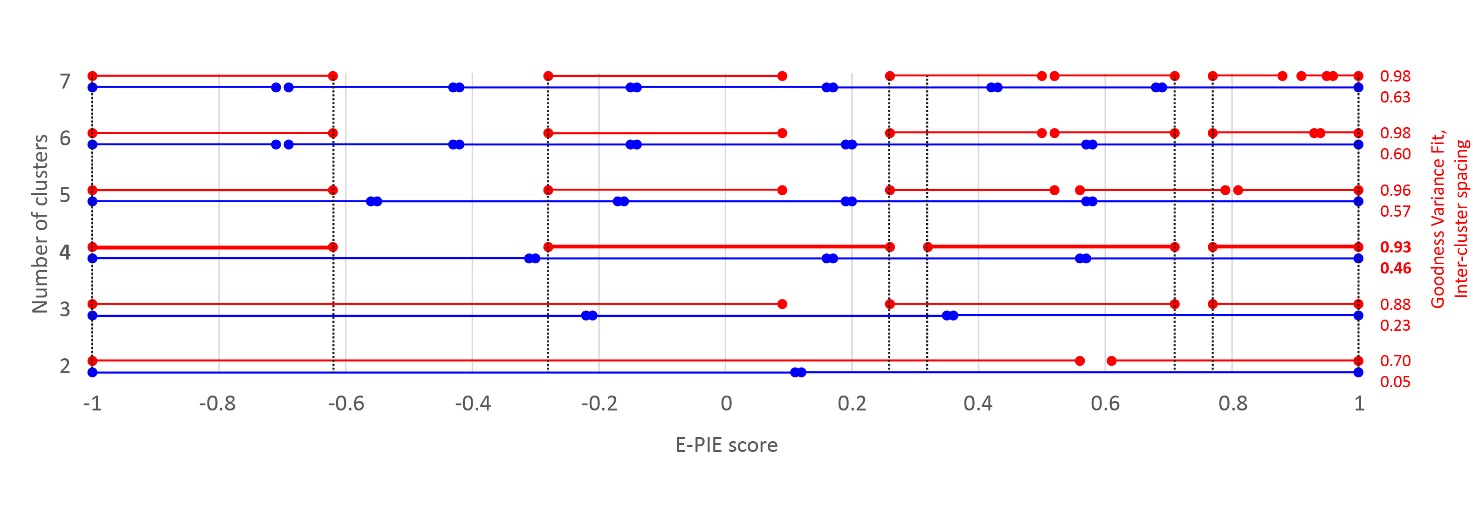


Figure S5: Cluster boundaries using Jenks natural breaks^27^ of two- to seven-cluster optimisations for the population of *HiQuake* cases (red, with the chosen four-cluster optimisation shown by heavy line) and the random subset population of scores (as in S3b & S4) taken from all possible permutations of *E-PIE* (blue). Vertical black dashed lines highlight the break points of the chosen four-cluster optimisation in relation to the other solutions. Goodness Variance Fit and inter-cluster spacing (red text) shown for the *HiQuake* cluster solutions.

Table S6: Logical implications of answer choices for the nine Questions posed in *E-PIE*. Natural-R: natural origin required, Induced-R: human-induced origin required, Either-P: either natural or human-induced origin permitted^16^.

| Question number | Answer | | |
| --- | --- | --- | --- |
|  | b | c | d |
| 1 | Natural-R | Either-P | Either-P |
| 2 | Natural-R | Either-P | Either-P |
| 3 | Natural-R | Either-P | Either-P |
| 4 | Either-P | Either-P | Induced-R |
| 5 | Either-P | Either-P | Either-P |
| 6 | Either-P | Either-P | Either-P |
| 7 | Natural-R | Either-P | Induced-R |
| 8 | Either-P | Either-P | Either-P |
| 9 | Either-P | Either-P | Either-P |

Table S7: The assessment results used in the analysis: *E-PIE* scores (9 questions) of each of the cases in *HiQuake,* conducted by a single assessor. a = no data, b = natural, c = equivocal, d = induced, x = no data across all questions. The results are provided in the attached data file ‘Assessment.csv’

| **ID** | **Earthquake Cause** | **Project Name** | **Q1** | **Q2** | **Q3** | **Q4** | **Q5** | **Q6** | **Q7** | **Q8** | **Q9** | **E-PIE score** |
| --- | --- | --- | --- | --- | --- | --- | --- | --- | --- | --- | --- | --- |
| 10001 | CCS | In Salah | d | d | d | d | a | a | a | d | d | 0.91 |
| 10002 | CCS | CCS1 well, Illinois Basin-Decatur Project | d | d | d | a | a | a | c | d | a | 0.61 |
| 10003 | CCS | CCS2 well, Illinois Industrial Carbon Capture and Sequestration Sources | d | d | d | c | b | b | a | c | a | 0.53 |
| 20001 | Chemical explosion | Tbilisi region | d | d | a | d | a | a | a | a | a | 0.58 |
| 30001 | Coal Bed Methane (CBM) | Selemo and Lesedi pilot pods | d | b | b | a | c | c | b | b | a | -0.58 |
| 40001 | Construction | Folkestone | d | d | a | a | d | d | a | a | a | 0.36 |
| 40002 | Construction | Taipei 101 | d | c | c | d | b | b | c | c | a | 0.25 |
| 50001 | Conventional Oil and Gas | Neftegorsk earthquake | d | d | a | a | a | a | a | d | a | 0.33 |
| 50002 | Conventional Oil and Gas | Kuzey (Northern) Marmara field, Thrace Basin | d | c | c | d | a | a | a | a | a | 0.30 |
| 50003 | Conventional Oil and Gas | Gazli | d | d | d | d | d | d | c | c | d | 0.94 |
| 50004 | Conventional Oil and Gas | Coalinga, California | d | d | c | a | a | a | c | b | a | 0.28 |
| 50005 | Conventional Oil and Gas | Long Beach earthquake (Huntington Beach oilfields), California | d | d | c | d | c | c | a | c | a | 0.58 |
| 50006 | Conventional Oil and Gas | Kettleman North Dome, California | d | d | b | b | c | c | c | c | a | -0.25 |
| 50007 | Conventional Oil and Gas | Barsa-Gelmes-Vishka | d | c | a | d | a | a | a | a | a | 0.30 |
| 50008 | Conventional Oil and Gas | Montebello (Whittier Narrows), California | d | d | c | a | c | c | c | d | a | 0.33 |
| 50009 | Conventional Oil and Gas | Shanul (Shanoul) gas field | d | d | d | a | c | c | c | c | a | 0.58 |
| 50010 | Conventional Oil and Gas | Caviaga, Po Valley | x | x | x | x | x | x | x | x | x |  |
| 50011 | Conventional Oil and Gas | Cogdell Field, Texas | d | d | d | d | a | a | a | c | a | 0.86 |
| 50012 | Conventional Oil and Gas | El Reno, Oklahoma | d | a | a | a | a | a | a | a | a | 0.03 |
| 50013 | Conventional Oil and Gas | San Ardo field, California | d | d | d | d | b | b | c | d | d | 0.86 |
| 50014 | Conventional Oil and Gas | Torrance oil field, California | d | d | d | d | b | a | c | c | a | 0.83 |
| 50015 | Conventional Oil and Gas | Wilmington, California | d | d | d | a | b | b | d | a | a | 0.55 |
| 50016 | Conventional Oil and Gas | Snipe Lake, Alberta | d | d | d | a | d | d | a | a | a | 0.64 |
| 50017 | Conventional Oil and Gas | Playa del Rey (Venice) oil field | d | d | a | d | a | a | a | a | a | 0.58 |
| 50018 | Conventional Oil and Gas | Panhandle field | d | d | a | a | a | a | a | a | a | 0.30 |
| 50019 | Conventional Oil and Gas | Inglewood and Hawthorne, California | d | d | d | d | c | c | a | d | a | 0.89 |
| 50020 | Conventional Oil and Gas | Richland County, Illinois | x | x | x | x | x | x | x | x | x |  |
| 50021 | Conventional Oil and Gas | Brewton (Big Escambia Creek, Little Rock, and Sizemore Creek fields), Alabama | d | d | d | b | c | c | c | a | a | 0.30 |
| 50022 | Conventional Oil and Gas | Fashing, Texas | d | d | d | b | d | a | a | a | d | 0.36 |
| 50023 | Conventional Oil and Gas | Dominguez oil field, California | d | d | d | d | c | a | a | a | a | 0.86 |
| 50024 | Conventional Oil and Gas | Santa Fe Springs oil field | d | d | d | a | a | a | a | a | a | 0.58 |
| 50025 | Conventional Oil and Gas | East Texas (Gladewater), Texas | d | d | a | a | a | a | a | a | a | 0.30 |
| 50026 | Conventional Oil and Gas | Starogroznenskoe | d | a | d | a | a | a | a | d | a | 0.33 |
| 50027 | Conventional Oil and Gas | Minagish/Umm Gudair oil fields (for largest eq) | d | d | d | a | c | a | a | a | a | 0.58 |
| 50028 | Conventional Oil and Gas | Catoosa, Oklahoma | x | x | x | x | x | x | x | x | x |  |
| 50029 | Conventional Oil and Gas | Renqiu | d | d | d | d | d | a | a | a | a | 0.89 |
| 50030 | Conventional Oil and Gas | Gudermes | x | x | x | x | x | x | x | x | x |  |
| 50031 | Conventional Oil and Gas | Rotenberg/Neuenkirchen and Söhlingen field (Rotenberg earthquake) | d | d | d | d | d | a | c | d | a | 0.91 |
| 50032 | Conventional Oil and Gas | Cogdell Field, Texas | d | d | a | d | b | a | c | a | a | 0.55 |
| 50033 | Conventional Oil and Gas | Eagle/Eagle West | d | d | d | d | d | a | a | a | a | 0.89 |
| 50034 | Conventional Oil and Gas | Poseidon Norte gas field | d | a | a | d | b | a | a | a | a | 0.28 |
| 50035 | Conventional Oil and Gas | Castor | d | d | d | d | d | d | c | d | a | 0.94 |
| 50036 | Conventional Oil and Gas | Ghawar | x | x | x | x | x | x | x | x | x |  |
| 50037 | Conventional Oil and Gas | Lacq (Arette) | d | d | d | a | c | a | a | d | d | 0.64 |
| 50038 | Conventional Oil and Gas | Wortham-Mexia, Texas | d | d | d | a | a | a | a | a | a | 0.58 |
| 50039 | Conventional Oil and Gas | Soltau field | x | x | x | x | x | x | x | x | x |  |
| 50040 | Conventional Oil and Gas | Romashkinskoye (Romashkino field), Volga-Ural | d | d | d | d | b | a | a | a | a | 0.83 |
| 50041 | Conventional Oil and Gas | Kermit, Texas | d | d | a | b | a | a | c | a | a | 0.03 |
| 50042 | Conventional Oil and Gas | Strachan, Alberta | x | x | x | x | x | x | x | x | x |  |
| 50043 | Conventional Oil and Gas | Beverly Hills, Santa Monica, California | x | x | x | x | x | x | x | x | x |  |
| 50044 | Conventional Oil and Gas | Lacq (Arette) | d | d | d | a | a | a | c | d | a | 0.61 |
| 50045 | Conventional Oil and Gas | Imogene (Pleasanton), Texas | d | d | a | b | b | a | a | a | a | 0.00 |
| 50046 | Conventional Oil and Gas | Alice (Stratton field), Texas | d | d | d | b | a | a | a | a | a | 0.30 |
| 50047 | Conventional Oil and Gas | Syke (Bassum, Niedersachsen) field | x | x | x | x | x | x | x | x | x |  |
| 50048 | Conventional Oil and Gas | Klosterseelte field | x | x | x | x | x | x | x | x | x |  |
| 50049 | Conventional Oil and Gas | Inglewood, California | d | d | d | a | b | a | a | a | a | 0.55 |
| 50050 | Conventional Oil and Gas | Falls City, Texas | d | d | a | b | b | a | a | a | d | 0.03 |
| 50051 | Conventional Oil and Gas | Tengiz field | d | a | a | a | d | a | a | a | a | 0.06 |
| 50052 | Conventional Oil and Gas | Azle/Reno,Texas | d | d | d | d | d | a | c | a | d | 0.91 |
| 50053 | Conventional Oil and Gas | Hunt, Alabama/Mississippi | x | x | x | x | x | x | x | x | x |  |
| 50054 | Conventional Oil and Gas | Hutubi, Southern Junggar Basin | d | d | d | d | b | a | a | a | d | 0.86 |
| 50055 | Conventional Oil and Gas | Bergermeer (Alkmaar) | d | d | d | a | d | a | a | a | a | 0.61 |
| 50056 | Conventional Oil and Gas | Keystone I&II, Texas | d | d | b | b | a | a | c | a | a | -0.25 |
| 50057 | Conventional Oil and Gas | East Durant, Oklahoma | x | x | x | x | x | x | x | x | x |  |
| 50058 | Conventional Oil and Gas | Orcutt, California | x | x | x | x | x | x | x | x | x |  |
| 50059 | Conventional Oil and Gas | Dollarhide, Texas/New Mexico | x | x | x | x | x | x | x | x | x |  |
| 50060 | Conventional Oil and Gas | Ward-Estes, Texas | x | x | x | x | x | x | x | x | x |  |
| 50061 | Conventional Oil and Gas | Coyote East and Richfield, Yorba Linga, California | x | x | x | x | x | x | x | x | x |  |
| 50062 | Conventional Oil and Gas | Roswinkel | d | d | d | b | d | a | a | a | a | 0.33 |
| 50063 | Conventional Oil and Gas | Groningen | d | d | d | d | d | a | a | a | a | 0.89 |
| 50064 | Conventional Oil and Gas | Gobles, Ontario | d | d | d | a | c | a | c | a | a | 0.58 |
| 50065 | Conventional Oil and Gas | North Panhandle (Lambert), Texas | x | x | x | x | x | x | x | x | x |  |
| 50066 | Conventional Oil and Gas | Grozny, Chechen Republic | a | a | a | d | a | a | a | a | a | 0.28 |
| 50067 | Conventional Oil and Gas | Horse Hill 1 (Newdigate earthquake sequence) | d | d | d | d | c | a | a | a | d | 0.89 |
| 50068 | Conventional Oil and Gas | Völkersen field | d | d | a | a | c | a | c | d | d | 0.36 |
| 50069 | Conventional Oil and Gas | War-Wink, Texas | x | x | x | x | x | x | x | x | x |  |
| 50070 | Conventional Oil and Gas | Ekofisk | d | d | d | a | b | a | c | a | d | 0.58 |
| 50071 | Conventional Oil and Gas | Ward-South, Texas | x | x | x | x | x | x | x | x | x |  |
| 50072 | Conventional Oil and Gas | Dora Roberts, Texas | x | x | x | x | x | x | x | x | x |  |
| 50073 | Conventional Oil and Gas | Monahans, Texas | x | x | x | x | x | x | x | x | x |  |
| 50074 | Conventional Oil and Gas | Visselhövede field | x | x | x | x | x | x | x | x | x |  |
| 50075 | Conventional Oil and Gas | Sleepy Hollow, Nebraska | x | x | x | x | x | x | x | x | x |  |
| 50076 | Conventional Oil and Gas | Assen | d | d | d | a | d | a | a | a | a | 0.61 |
| 50077 | Conventional Oil and Gas | Eleveld | x | x | x | x | x | x | x | x | x |  |
| 50078 | Conventional Oil and Gas | Langwedel field | x | x | x | x | x | x | x | x | x |  |
| 50079 | Conventional Oil and Gas | Love County, Oklahoma | x | x | x | x | x | x | x | x | x |  |
| 50080 | Conventional Oil and Gas | Middelie | x | x | x | x | x | x | x | x | x |  |
| 50081 | Conventional Oil and Gas | Bergen | x | x | x | x | x | x | x | x | x |  |
| 50082 | Conventional Oil and Gas | Zhanazhol field | d | d | a | a | a | a | a | a | a | 0.30 |
| 50083 | Conventional Oil and Gas | Verden field | x | x | x | x | x | x | x | x | x |  |
| 50084 | Conventional Oil and Gas | Annerveen | x | x | x | x | x | x | x | x | x |  |
| 50085 | Conventional Oil and Gas | Appelscha | x | x | x | x | x | x | x | x | x |  |
| 50086 | Conventional Oil and Gas | Dalen | x | x | x | x | x | x | x | x | x |  |
| 50087 | Conventional Oil and Gas | Roden | x | x | x | x | x | x | x | x | x |  |
| 50088 | Conventional Oil and Gas | Walsrode field | d | d | d | b | d | a | a | a | d | 0.36 |
| 50089 | Conventional Oil and Gas | Shuiba reservoir | d | d | d | b | c | a | a | a | a | 0.30 |
| 50090 | Conventional Oil and Gas | Emmen | x | x | x | x | x | x | x | x | x |  |
| 50091 | Conventional Oil and Gas | Apollo-Hendrick, Texas | d | d | d | d | c | a | c | a | a | 0.86 |
| 50092 | Conventional Oil and Gas | Cold Lake, Alberta | x | x | x | x | x | x | x | x | x |  |
| 50093 | Conventional Oil and Gas | Lacq (Arette) | d | d | d | d | d | a | a | d | a | 0.91 |
| 50094 | Conventional Oil and Gas | VriesNoord | x | x | x | x | x | x | x | x | x |  |
| 50095 | Conventional Oil and Gas | New Harmony, Indiana | a | d | d | a | c | a | a | d | a | 0.58 |
| 50096 | Conventional Oil and Gas | Emmen-Nieuw Amsterdam | x | x | x | x | x | x | x | x | x |  |
| 50097 | Conventional Oil and Gas | Příbram (Háje) | a | d | d | a | c | a | a | a | a | 0.55 |
| 50098 | Conventional Oil and Gas | Schoonebeek | x | x | x | x | x | x | x | x | x |  |
| 50099 | Conventional Oil and Gas | Coevorden | x | x | x | x | x | x | x | x | x |  |
| 50100 | Conventional Oil and Gas | Ureterp | x | x | x | x | x | x | x | x | x |  |
| 50101 | Conventional Oil and Gas | VriesCentraal | x | x | x | x | x | x | x | x | x |  |
| 50102 | Conventional Oil and Gas | Seventy Six oil field, Clinton County, Kentucky | d | d | d | d | c | a | c | d | a | 0.89 |
| 50103 | Conventional Oil and Gas | Grane oil field (Well 25/11‐G‐8 A) | d | a | a | d | a | a | a | a | a | 0.30 |
| 50104 | Conventional Oil and Gas | Kirby Misperton field | x | x | x | x | x | x | x | x | x |  |
| 50105 | Conventional Oil and Gas | Bergermeer | d | a | a | d | a | a | a | a | d | 0.33 |
| 50106 | Conventional Oil and Gas | Hoadley gas field | d | a | a | a | a | a | a | a | a | 0.03 |
| 50107 | Conventional Oil and Gas | Pembina oil field | d | a | a | a | a | a | a | a | a | 0.03 |
| 50108 | Conventional Oil and Gas | Weyburn, Saskatchewan | d | d | d | c | a | a | a | a | d | 0.61 |
| 50109 | Conventional Oil and Gas | Tazlau | d | d | d | a | a | a | a | a | a | 0.58 |
| 50110 | Conventional Oil and Gas | Novo-Elkhovskoye, Volga-Ural | x | x | x | x | x | x | x | x | x |  |
| 50111 | Conventional Oil and Gas | South Houston, Texas | x | x | x | x | x | x | x | x | x |  |
| 50112 | Conventional Oil and Gas | Clinton, Texas | x | x | x | x | x | x | x | x | x |  |
| 50113 | Conventional Oil and Gas | MyKawa, Texas | x | x | x | x | x | x | x | x | x |  |
| 50114 | Conventional Oil and Gas | Blue Ridge, Texas | x | x | x | x | x | x | x | x | x |  |
| 50115 | Conventional Oil and Gas | Webster, Texas | x | x | x | x | x | x | x | x | x |  |
| 50116 | Conventional Oil and Gas | Goose Creek, Texas | x | x | x | x | x | x | x | x | x |  |
| 50117 | Conventional Oil and Gas | Costa Oriental, Lake Maracaibo | x | x | x | x | x | x | x | x | x |  |
| 50118 | Conventional Oil and Gas | Austin Chalk, Giddings Field, Texas | d | d | d | d | a | a | a | a | a | 0.86 |
| 50119 | Conventional Oil and Gas | Shengli, Shandong Province | x | x | x | x | x | x | x | x | x |  |
| 50120 | Conventional Oil and Gas | Dan | x | x | x | x | x | x | x | x | x |  |
| 50121 | Conventional Oil and Gas | South Eugene Island, Louisianna | x | x | x | x | x | x | x | x | x |  |
| 50122 | Conventional Oil and Gas | Meillon | x | x | x | x | x | x | x | x | x |  |
| 50123 | Conventional Oil and Gas | Vishund | x | x | x | x | x | x | x | x | x |  |
| 50124 | Conventional Oil and Gas | Valhall | d | d | d | a | a | a | c | a | d | 0.61 |
| 50125 | Conventional Oil and Gas | Norg | x | x | x | x | x | x | x | x | x |  |
| 50126 | Conventional Oil and Gas | Grijpskerk | x | x | x | x | x | x | x | x | x |  |
| 50127 | Conventional Oil and Gas | Samgori-Ninotsminda (Tbilisis hydrothermal area) | x | x | x | x | x | x | x | x | x |  |
| 50128 | Conventional Oil and Gas | Goldenstedt field | d | d | d | b | d | a | a | a | d | 0.36 |
| 50129 | Conventional Oil and Gas | Hemmelte field | d | d | d | b | d | a | a | a | d | 0.36 |
| 50130 | Conventional Oil and Gas | Dethlingen field | d | d | d | b | d | a | a | a | d | 0.36 |
| 50131 | Conventional Oil and Gas | Barrien field | d | d | d | b | d | a | a | a | d | 0.36 |
| 50132 | Conventional Oil and Gas | Burgmoor field | d | d | d | b | d | a | a | a | d | 0.36 |
| 50133 | Conventional Oil and Gas | Husum field | d | d | d | b | d | a | a | a | d | 0.36 |
| 50134 | Conventional Oil and Gas | Santa Barbara, California | x | x | x | x | x | x | x | x | x |  |
| 50135 | Conventional Oil and Gas | Ventura, California | x | x | x | x | x | x | x | x | x |  |
| 50136 | Conventional Oil and Gas | Kern County, California | x | x | x | x | x | x | x | x | x |  |
| 60001 | Deep penetrating bombs | Kosovo | c | c | c | a | c | a | a | a | a | 0.00 |
| 60002 | Deep penetrating bombs | Baghdad | c | b | a | b | c | a | a | a | a | -0.55 |
| 60003 | Deep penetrating bombs | Tora Bora | d | d | a | a | b | a | a | d | a | 0.30 |
| 60004 | Deep penetrating bombs | Kirkuk | c | c | a | a | c | a | a | a | a | 0.00 |
| 70001 | Fracking | N201-H24 well pad, Changning shale gas block, Xingwen County, Sichuan Province | d | d | d | a | a | a | c | d | a | 0.61 |
| 70002 | Fracking | N201-H18 well pad, Changning shale gas block, Xingwen County, Sichuan Province | d | d | d | d | a | a | c | d | a | 0.89 |
| 70003 | Fracking | H7 well pad, Shangluo site, Zhaotong field | d | d | d | c | c | c | c | d | a | 0.61 |
| 70004 | Fracking | 116 km WNW of Fort St. John (well 10 in Mahani et al., 2017) | d | d | d | d | c | c | c | d | a | 0.89 |
| 70005 | Fracking | Rongxian County, Sichuan Basin | d | d | d | a | c | a | c | c | a | 0.58 |
| 70006 | Fracking | 16 km SW Fort St. John | a | d | d | d | a | a | a | a | a | 0.83 |
| 70007 | Fracking | Northern Montney Earthquake, British Columbia | x | x | x | x | x | x | x | x | x |  |
| 70008 | Fracking | Septimus (Montney Trend) | d | d | a | d | a | a | a | a | a | 0.58 |
| 70009 | Fracking | Texas (Cluster C) | d | d | d | d | d | d | a | a | a | 0.91 |
| 70010 | Fracking | Duvernay East Shale Basin 10 | d | d | d | d | d | a | c | d | a | 0.89 |
| 70011 | Fracking | Fox Creek (SS17) | d | d | d | d | b | a | c | d | a | 0.86 |
| 70012 | Fracking | Fox Creek (SS6) Alberta (Waskahigan and McKinley fields) (Well Pad 1) | d | d | d | d | b | a | c | a | a | 0.83 |
| 70013 | Fracking | Fox Creek (SS10) | d | d | d | d | b | a | c | d | a | 0.86 |
| 70014 | Fracking | Horn River Basin | d | d | d | d | d | d | a | d | a | 0.94 |
| 70015 | Fracking | Wolf 2H well, Noble County, Ohio | d | a | a | a | c | c | a | a | a | 0.03 |
| 70016 | Fracking | Fox Creek (SS7) (Well Pad 3) | d | d | d | d | b | a | c | d | a | 0.86 |
| 70017 | Fracking | Beg-Town (Montney Trend) | d | d | d | d | a | a | a | a | a | 0.86 |
| 70018 | Fracking | Weiyuan site | x | x | x | x | x | x | x | x | x |  |
| 70019 | Fracking | Fox Creek (SS1, SS4) | d | d | d | d | b | a | c | d | a | 0.86 |
| 70020 | Fracking | Tony Creek dual Microseismic Experiment (ToC2ME), Fox Creek, Alberta | d | d | d | d | a | a | c | c | a | 0.86 |
| 70021 | Fracking | Caribou (Montney Trend) | d | d | d | d | a | a | a | a | a | 0.86 |
| 70022 | Fracking | Eagleton 1-29, Oklahoma | d | d | d | d | b | b | a | c | a | 0.80 |
| 70023 | Fracking | Fox Creek (SS3) | d | d | a | d | b | a | a | a | a | 0.55 |
| 70024 | Fracking | Fox Creek (SS5) | d | c | a | d | b | a | a | a | a | 0.28 |
| 70025 | Fracking | Fox Creek (SS9) (Well Pad 6) | d | d | a | d | b | a | c | a | a | 0.55 |
| 70026 | Fracking | Fox Creek (SS12) | d | d | a | d | b | a | c | a | a | 0.55 |
| 70027 | Fracking | Love County region, Oklahoma | d | d | d | d | c | a | a | d | a | 0.58 |
| 70028 | Fracking | Coal County region, Oklahoma | d | d | d | d | c | a | a | a | a | 0.61 |
| 70029 | Fracking | Blaine County region, Oklahoma | d | d | d | d | c | a | a | d | a | 0.33 |
| 70030 | Fracking | Fox Creek (SS2) | d | d | a | d | b | a | a | a | a | 0.55 |
| 70031 | Fracking | Duvernay East Shale Basin 02 | d | d | d | d | a | a | a | d | a | 0.89 |
| 70032 | Fracking | Fox Creek (SS14) | d | d | a | d | b | a | b | c | a | 0.53 |
| 70033 | Fracking | Kay County region, Oklahoma | d | d | d | c | b | a | a | c | a | 0.28 |
| 70034 | Fracking | Major County region, Oklahoma | d | d | a | c | a | a | a | c | a | 0.30 |
| 70035 | Fracking | Donato well, Monroe County, Ohio | d | d | d | d | d | a | c | c | a | 0.89 |
| 70036 | Fracking | Cardston, Alberta (Ninastoko field) | d | d | d | d | c | c | c | d | d | 0.91 |
| 70037 | Fracking | Poland Township, Ohio | d | d | d | d | d | d | c | d | a | 0.94 |
| 70038 | Fracking | Fox Creek (SS16) | d | d | a | d | c | a | c | a | a | 0.58 |
| 70039 | Fracking | Kay County region, Oklahoma | d | d | d | c | b | a | a | c | a | 0.28 |
| 70040 | Fracking | Blaine County region, Oklahoma | d | d | d | d | c | a | a | d | a | 0.33 |
| 70041 | Fracking | North Grady County region, Oklahoma | d | d | d | d | c | a | a | c | a | 0.58 |
| 70042 | Fracking | Woodward County region, Oklahoma | d | d | d | d | c | a | a | a | a | 0.58 |
| 70043 | Fracking | Pittsburgh County region, Oklahoma | d | d | d | d | c | a | a | d | a | 0.61 |
| 70044 | Fracking | Eola-Robberson field, Oklahoma | d | d | d | d | d | d | a | d | a | 0.94 |
| 70045 | Fracking | Fox Creek (SS8) (Well Pad 2) | d | d | a | d | c | a | c | d | a | 0.61 |
| 70046 | Fracking | Fox Creek (Well Pad 5) | d | d | a | d | c | a | a | a | a | 0.58 |
| 70047 | Fracking | Fox Creek (SS11) | d | d | d | d | c | c | c | d | a | 0.89 |
| 70048 | Fracking | Fox Creek (SS13) | d | d | a | a | c | a | c | a | a | 0.30 |
| 70049 | Fracking | Love County region, Oklahoma | d | d | d | d | c | a | a | d | a | 0.86 |
| 70050 | Fracking | McClain County region, Oklahoma | d | d | d | d | c | a | a | d | a | 0.89 |
| 70051 | Fracking | Major County region, Oklahoma | d | d | d | d | a | a | a | a | a | 0.86 |
| 70052 | Fracking | Preston New Road | x | x | x | x | x | x | x | x | x |  |
| 70053 | Fracking | Doe-Dawson (Montney Trend) | d | d | d | d | d | d | a | a | a | 0.91 |
| 70054 | Fracking | Fox Creek (SS15) | d | d | a | d | b | a | d | a | a | 0.58 |
| 70055 | Fracking | Love County region, Oklahoma | d | d | d | d | c | a | a | d | a | 0.58 |
| 70056 | Fracking | McClain County region, Oklahoma | d | d | d | d | c | a | a | d | a | 0.86 |
| 70057 | Fracking | Coal County region, Oklahoma | d | d | d | d | c | a | a | a | a | 0.86 |
| 70058 | Fracking | Coal County region, Oklahoma | d | d | d | d | c | a | a | a | a | 0.86 |
| 70059 | Fracking | Kay County region, Oklahoma | d | d | d | c | b | a | a | c | a | 0.86 |
| 70060 | Fracking | Kingfisher County region, Oklahoma | d | d | d | d | c | a | a | a | a | 0.86 |
| 70061 | Fracking | Kingfisher County region, Oklahoma | d | d | d | d | c | a | a | a | a | 0.86 |
| 70062 | Fracking | Kingfisher County region, Oklahoma | d | d | d | d | c | a | a | a | a | 0.86 |
| 70063 | Fracking | Major County region, Oklahoma | d | d | d | d | c | a | a | a | a | 0.86 |
| 70064 | Fracking | North Canadian County region, Oklahoma | d | d | d | d | c | a | a | a | a | 0.86 |
| 70065 | Fracking | South Canadian County region, Oklahoma | d | d | d | d | c | a | a | a | a | 0.58 |
| 70066 | Fracking | North Grady County region, Oklahoma | d | d | d | d | c | a | a | c | a | 0.58 |
| 70067 | Fracking | Marshall County region, Oklahoma | d | d | d | c | c | a | a | a | a | 0.58 |
| 70068 | Fracking | North Stephens County region, Oklahoma | d | d | d | d | c | a | a | a | a | 0.58 |
| 70069 | Fracking | Gilmer County, West Virginia | d | d | d | a | c | a | a | d | a | 0.61 |
| 70070 | Fracking | Conotton well, Harrison County, Ohio | d | d | d | a | a | a | a | d | a | 0.61 |
| 70071 | Fracking | Fox Creek (Well Pad 4) | d | d | d | d | c | a | a | a | a | 0.58 |
| 70072 | Fracking | Blaine County region, Oklahoma | d | d | d | d | c | a | a | d | a | 0.61 |
| 70073 | Fracking | South Grady County region, Oklahoma | d | d | d | d | c | a | a | a | a | 0.58 |
| 70074 | Fracking | Carter County region, Oklahoma | d | d | d | d | c | a | a | d | a | 0.61 |
| 70075 | Fracking | North Stephens County region, Oklahoma | d | d | d | d | c | a | a | a | a | 0.58 |
| 70076 | Fracking | Duvernay East Shale Basin 01 | d | d | d | d | d | a | c | d | a | 0.89 |
| 70077 | Fracking | Kirkwood A wells, Belmont County, Ohio | d | d | d | d | d | d | a | d | a | 0.94 |
| 70078 | Fracking | Hamilton well, Harrison County, Ohio | d | d | d | c | d | d | c | c | a | 0.64 |
| 70079 | Fracking | McClain County region, Oklahoma | d | d | d | d | c | a | a | d | a | 0.89 |
| 70080 | Fracking | McClain County region, Oklahoma | d | d | d | d | c | a | a | d | a | 0.89 |
| 70081 | Fracking | Coal County region, Oklahoma | d | d | d | d | c | a | a | a | a | 0.86 |
| 70082 | Fracking | South Canadian County region, Oklahoma | d | d | d | d | c | a | a | a | a | 0.86 |
| 70083 | Fracking | North Grady County region, Oklahoma | d | d | d | d | c | a | a | c | a | 0.86 |
| 70084 | Fracking | South Grady County region, Oklahoma | d | d | d | d | c | a | a | a | a | 0.86 |
| 70085 | Fracking | Carter County region, Oklahoma | d | d | d | d | c | a | a | d | a | 0.89 |
| 70086 | Fracking | Love County region, Oklahoma | d | d | d | d | c | a | a | d | a | 0.89 |
| 70087 | Fracking | Coal County region, Oklahoma | d | d | d | d | c | a | a | a | a | 0.86 |
| 70088 | Fracking | Southern Stephens County region, Oklahoma | d | d | d | d | c | a | a | a | a | 0.86 |
| 70089 | Fracking | Southern Stephens County region, Oklahoma | d | d | d | d | c | a | a | a | a | 0.86 |
| 70090 | Fracking | North Canadian County region, Oklahoma | d | d | d | d | c | a | a | a | a | 0.86 |
| 70091 | Fracking | Garvin County region, Oklahoma | d | d | d | d | c | a | a | a | a | 0.86 |
| 70092 | Fracking | Kay County region, Oklahoma | d | d | d | c | b | a | a | c | a | 0.55 |
| 70093 | Fracking | Blaine County region, Oklahoma | d | d | d | d | c | a | a | d | a | 0.89 |
| 70094 | Fracking | North Grady County region, Oklahoma | d | d | d | d | c | a | a | c | a | 0.86 |
| 70095 | Fracking | North Stephens County region, Oklahoma | d | d | d | d | c | a | a | a | a | 0.86 |
| 70096 | Fracking | Pittsburgh County region, Oklahoma | d | d | d | d | c | a | a | d | a | 0.89 |
| 70097 | Fracking | Wheeler well, Guernsey County, Ohio | d | d | d | d | c | a | c | d | a | 0.89 |
| 70098 | Fracking | Preese Hall | d | d | d | d | a | a | a | a | a | 0.86 |
| 70099 | Fracking | McClain County region, Oklahoma | d | d | d | d | c | a | a | d | a | 0.89 |
| 70100 | Fracking | North Canadian County region, Oklahoma | d | d | d | d | c | a | a | a | a | 0.86 |
| 70101 | Fracking | South Grady County region, Oklahoma | d | d | d | d | c | a | a | a | a | 0.86 |
| 70102 | Fracking | South Grady County region, Oklahoma | d | d | d | d | c | a | a | a | a | 0.86 |
| 70103 | Fracking | Carter County region, Oklahoma | d | d | d | d | c | a | a | d | a | 0.89 |
| 70104 | Fracking | Altares (Montney Trend) | d | d | d | a | a | a | a | a | a | 0.58 |
| 70105 | Fracking | Ryser well, Harrison County, Ohio | d | d | d | b | d | d | c | b | a | 0.33 |
| 70106 | Fracking | Love County region, Oklahoma | d | d | d | d | c | a | a | d | a | 0.89 |
| 70107 | Fracking | Love County region, Oklahoma | d | d | d | d | c | a | a | d | a | 0.89 |
| 70108 | Fracking | McClain County region, Oklahoma | d | d | d | d | c | a | a | d | a | 0.89 |
| 70109 | Fracking | Major County region, Oklahoma | d | d | d | d | c | a | a | a | a | 0.86 |
| 70110 | Fracking | Blaine County region, Oklahoma | d | d | d | d | c | a | a | d | a | 0.89 |
| 70111 | Fracking | North Grady County region, Oklahoma | d | d | d | d | c | a | a | c | a | 0.86 |
| 70112 | Fracking | Woodward County region, Oklahoma | d | d | d | d | c | a | a | a | a | 0.86 |
| 70113 | Fracking | Carter County region, Oklahoma | d | d | d | d | c | a | a | d | a | 0.89 |
| 70114 | Fracking | Marshall County region, Oklahoma | d | d | d | c | c | a | a | a | a | 0.58 |
| 70115 | Fracking | Pittsburgh County region, Oklahoma | d | d | d | d | c | a | a | d | a | 0.89 |
| 70116 | Fracking | Duvernay East Shale Basin 02 | d | d | d | d | d | a | c | d | a | 0.91 |
| 70117 | Fracking | Love County region, Oklahoma | d | d | d | d | c | a | a | d | a | 0.89 |
| 70118 | Fracking | Love County region, Oklahoma | d | d | d | d | c | a | a | d | a | 0.89 |
| 70119 | Fracking | Kay County region, Oklahoma | d | d | d | c | b | a | a | c | a | 0.55 |
| 70120 | Fracking | North Canadian County region, Oklahoma | d | d | d | d | c | a | a | a | a | 0.86 |
| 70121 | Fracking | Carter County region, Oklahoma | d | d | d | d | c | a | a | d | a | 0.89 |
| 70122 | Fracking | Garvin County region, Oklahoma | d | d | d | d | c | a | a | a | a | 0.86 |
| 70123 | Fracking | Pittsburgh County region, Oklahoma | d | d | d | d | c | a | a | d | a | 0.89 |
| 70124 | Fracking | Duvernay East Shale Basin 01 | d | d | d | d | d | a | c | d | a | 0.91 |
| 70125 | Fracking | Love County region, Oklahoma | d | d | d | d | c | a | a | d | a | 0.89 |
| 70126 | Fracking | Love County region, Oklahoma | d | d | d | d | c | a | a | d | a | 0.89 |
| 70127 | Fracking | Love County region, Oklahoma | d | d | d | d | c | a | a | d | a | 0.89 |
| 70128 | Fracking | Southern Stephens County region, Oklahoma | d | d | d | d | c | a | a | a | a | 0.86 |
| 70129 | Fracking | Kingfisher County region, Oklahoma | d | d | d | d | c | a | a | a | a | 0.86 |
| 70130 | Fracking | Kingfisher County region, Oklahoma | d | d | d | d | c | a | a | a | a | 0.86 |
| 70131 | Fracking | South Canadian County region, Oklahoma | d | d | d | d | c | a | a | a | a | 0.86 |
| 70132 | Fracking | North Grady County region, Oklahoma | d | d | d | d | c | a | a | c | a | 0.86 |
| 70133 | Fracking | South Grady County region, Oklahoma | d | d | d | d | c | a | a | a | a | 0.86 |
| 70134 | Fracking | Carter County region, Oklahoma | d | d | d | d | c | a | a | d | a | 0.89 |
| 70135 | Fracking | Duvernay East Shale Basin 09 | d | d | d | d | d | a | c | d | a | 0.91 |
| 70136 | Fracking | Bienville Parish, Louisiana | d | d | d | a | c | a | a | c | a | 0.58 |
| 70137 | Fracking | Edinburg (North Beaver Township), Pennsylvania | d | d | d | d | c | a | a | a | a | 0.86 |
| 70138 | Fracking | North Grady County region, Oklahoma | d | d | d | d | c | a | a | c | a | 0.86 |
| 70139 | Fracking | Tarbert well, Harrison County, Ohio | d | d | d | d | c | a | a | d | a | 0.89 |
| 70140 | Fracking | Carter County region, Oklahoma | d | d | d | d | c | a | a | d | a | 0.89 |
| 70141 | Fracking | North Grady County region, Oklahoma | d | d | d | d | c | a | a | c | a | 0.86 |
| 70142 | Fracking | Duvernay East Shale Basin 07&08 | d | d | d | d | d | a | c | d | a | 0.91 |
| 70143 | Fracking | South Canadian County region, Oklahoma | d | d | d | d | c | a | a | a | a | 0.86 |
| 70144 | Fracking | North Grady County region, Oklahoma | d | d | d | d | c | a | a | c | a | 0.86 |
| 70145 | Fracking | North Grady County region, Oklahoma | d | d | d | d | c | a | a | c | a | 0.86 |
| 70146 | Fracking | Davidson well, Harrison County, Ohio | d | d | d | d | d | a | a | c | a | 0.89 |
| 70147 | Fracking | Wysin Site (Well 3H) | d | d | d | c | d | a | a | a | b | 0.58 |
| 70148 | Fracking | Duvernay East Shale Basin 04 | d | d | d | d | d | a | c | d | a | 0.91 |
| 70149 | Fracking | Duvernay East Shale Basin 03 | d | d | d | d | d | a | c | d | a | 0.91 |
| 70150 | Fracking | Vozar well, Harrison County, Ohio | d | d | d | c | d | a | a | d | a | 0.64 |
| 70151 | Fracking | Wysin Site (Well 2H) | d | d | d | c | d | a | a | a | b | 0.58 |
| 70152 | Fracking | Horn River Basin | d | a | a | d | a | a | a | d | a | 0.33 |
| 70153 | Fracking | Cotton Valley, Texas | d | d | d | d | a | a | a | d | a | 0.89 |
| 70154 | Fracking | Montney Trend | d | d | d | d | a | a | d | d | d | 0.94 |
| 70155 | Fracking | Jonah field, Wyoming | d | d | a | d | a | a | a | d |  | 0.61 |
| 70156 | Fracking | Hughes County, Oklahoma | d | d | d | a | a | a | a | a | a | 0.58 |
| 70157 | Fracking | Western Canada | d | d | d | a | a | a | a | d | a | 0.61 |
| 70158 | Fracking | McClain County region, Oklahoma | d | d | d | d | c | a | a | d | a | 0.89 |
| 70159 | Fracking | McClain County region, Oklahoma | d | d | d | d | c | a | a | d | a | 0.89 |
| 70160 | Fracking | McClain County region, Oklahoma | d | d | d | d | c | a | a | d | a | 0.89 |
| 70161 | Fracking | McClain County region, Oklahoma | d | d | d | d | c | a | a | d | a | 0.89 |
| 70162 | Fracking | McClain County region, Oklahoma | d | d | d | d | c | a | a | d | a | 0.89 |
| 70163 | Fracking | McClain County region, Oklahoma | d | d | d | d | c | a | a | d | a | 0.89 |
| 70164 | Fracking | McClain County region, Oklahoma | d | d | d | d | c | a | a | d | a | 0.89 |
| 70165 | Fracking | McClain County region, Oklahoma | d | d | d | d | c | a | a | d | a | 0.89 |
| 70166 | Fracking | McClain County region, Oklahoma | d | d | d | d | c | a | a | d | a | 0.89 |
| 70167 | Fracking | McClain County region, Oklahoma | d | d | d | d | c | a | a | d | a | 0.89 |
| 70168 | Fracking | McClain County region, Oklahoma | d | d | d | d | c | a | a | d | a | 0.89 |
| 70169 | Fracking | McClain County region, Oklahoma | d | d | d | d | c | a | a | d | a | 0.89 |
| 70170 | Fracking | McClain County region, Oklahoma | d | d | d | d | c | a | a | d | a | 0.89 |
| 70171 | Fracking | McClain County region, Oklahoma | d | d | d | d | c | a | a | d | a | 0.89 |
| 70172 | Fracking | Coal County region, Oklahoma | d | d | d | d | c | a | a | a | a | 0.86 |
| 70173 | Fracking | Coal County region, Oklahoma | d | d | d | d | c | a | a | a | a | 0.86 |
| 70174 | Fracking | Coal County region, Oklahoma | d | d | d | d | c | a | a | a | a | 0.86 |
| 70175 | Fracking | Coal County region, Oklahoma | d | d | d | d | c | a | a | a | a | 0.86 |
| 70176 | Fracking | Coal County region, Oklahoma | d | d | d | d | c | a | a | a | a | 0.86 |
| 70177 | Fracking | Coal County region, Oklahoma | d | d | d | d | c | a | a | a | a | 0.86 |
| 70178 | Fracking | Coal County region, Oklahoma | d | d | d | d | c | a | a | a | a | 0.86 |
| 70179 | Fracking | Coal County region, Oklahoma | d | d | d | d | c | a | a | a | a | 0.86 |
| 70180 | Fracking | Coal County region, Oklahoma | d | d | d | d | c | a | a | a | a | 0.86 |
| 70181 | Fracking | Coal County region, Oklahoma | d | d | d | d | c | a | a | a | a | 0.86 |
| 70182 | Fracking | Coal County region, Oklahoma | d | d | d | d | c | a | a | a | a | 0.86 |
| 70183 | Fracking | Southern Stephens County region, Oklahoma | d | d | d | d | c | a | a | a | a | 0.86 |
| 70184 | Fracking | Southern Stephens County region, Oklahoma | d | d | d | d | c | a | a | a | a | 0.86 |
| 70185 | Fracking | Southern Stephens County region, Oklahoma | d | d | d | d | c | a | a | a | a | 0.86 |
| 70186 | Fracking | Southern Stephens County region, Oklahoma | d | d | d | d | c | a | a | a | a | 0.86 |
| 70187 | Fracking | Southern Stephens County region, Oklahoma | d | d | d | d | c | a | a | a | a | 0.86 |
| 70188 | Fracking | Southern Stephens County region, Oklahoma | d | d | d | d | c | a | a | a | a | 0.86 |
| 70189 | Fracking | Southern Stephens County region, Oklahoma | d | d | d | d | c | a | a | a | a | 0.86 |
| 70190 | Fracking | Southern Stephens County region, Oklahoma | d | d | d | d | c | a | a | a | a | 0.86 |
| 70191 | Fracking | Southern Stephens County region, Oklahoma | d | d | d | d | c | a | a | a | a | 0.86 |
| 70192 | Fracking | Southern Stephens County region, Oklahoma | d | d | d | d | c | a | a | a | a | 0.86 |
| 70193 | Fracking | Southern Stephens County region, Oklahoma | d | d | d | d | c | a | a | a | a | 0.86 |
| 70194 | Fracking | Southern Stephens County region, Oklahoma | d | d | d | d | c | a | a | a | a | 0.86 |
| 70195 | Fracking | Kay County region, Oklahoma | d | d | d | c | b | a | a | c | a | 0.55 |
| 70196 | Fracking | Kay County region, Oklahoma | d | d | d | c | b | a | a | c | a | 0.55 |
| 70197 | Fracking | Kay County region, Oklahoma | d | d | d | c | b | a | a | c | a | 0.55 |
| 70198 | Fracking | Kay County region, Oklahoma | d | d | d | c | b | a | a | c | a | 0.55 |
| 70199 | Fracking | Kay County region, Oklahoma | d | d | d | c | b | a | a | c | a | 0.55 |
| 70200 | Fracking | Kay County region, Oklahoma | d | d | d | c | b | a | a | c | a | 0.55 |
| 70201 | Fracking | Kay County region, Oklahoma | d | d | d | c | b | a | a | c | a | 0.55 |
| 70202 | Fracking | Kay County region, Oklahoma | d | d | d | c | b | a | a | c | a | 0.55 |
| 70203 | Fracking | Kay County region, Oklahoma | d | d | d | c | b | a | a | c | a | 0.55 |
| 70204 | Fracking | Kay County region, Oklahoma | d | d | d | c | b | a | a | c | a | 0.55 |
| 70205 | Fracking | Kay County region, Oklahoma | d | d | d | c | b | a | a | c | a | 0.55 |
| 70206 | Fracking | Kay County region, Oklahoma | d | d | d | c | b | a | a | c | a | 0.55 |
| 70207 | Fracking | Kay County region, Oklahoma | d | d | d | c | b | a | a | c | a | 0.55 |
| 70208 | Fracking | Kay County region, Oklahoma | d | d | d | c | b | a | a | c | a | 0.55 |
| 70209 | Fracking | Kingfisher County region, Oklahoma | d | d | d | d | c | a | a | a | a | 0.86 |
| 70210 | Fracking | Kingfisher County region, Oklahoma | d | d | d | d | c | a | a | a | a | 0.86 |
| 70211 | Fracking | Kingfisher County region, Oklahoma | d | d | d | d | c | a | a | a | a | 0.86 |
| 70212 | Fracking | Kingfisher County region, Oklahoma | d | d | d | d | c | a | a | a | a | 0.86 |
| 70213 | Fracking | Kingfisher County region, Oklahoma | d | d | d | d | c | a | a | a | a | 0.86 |
| 70214 | Fracking | Kingfisher County region, Oklahoma | d | d | d | d | c | a | a | a | a | 0.86 |
| 70215 | Fracking | Kingfisher County region, Oklahoma | d | d | d | d | c | a | a | a | a | 0.86 |
| 70216 | Fracking | Kingfisher County region, Oklahoma | d | d | d | d | c | a | a | a | a | 0.86 |
| 70217 | Fracking | Kingfisher County region, Oklahoma | d | d | d | d | c | a | a | a | a | 0.86 |
| 70218 | Fracking | Kingfisher County region, Oklahoma | d | d | d | d | c | a | a | a | a | 0.86 |
| 70219 | Fracking | Kingfisher County region, Oklahoma | d | d | d | d | c | a | a | a | a | 0.86 |
| 70220 | Fracking | Major County region, Oklahoma | d | d | d | d | c | a | a | a | a | 0.86 |
| 70221 | Fracking | Major County region, Oklahoma | d | d | d | d | c | a | a | a | a | 0.86 |
| 70222 | Fracking | Major County region, Oklahoma | d | d | d | d | c | a | a | a | a | 0.86 |
| 70223 | Fracking | Major County region, Oklahoma | d | d | d | d | c | a | a | a | a | 0.86 |
| 70224 | Fracking | Blaine County region, Oklahoma | d | d | d | d | c | a | a | d | a | 0.89 |
| 70225 | Fracking | Blaine County region, Oklahoma | d | d | d | d | c | a | a | d | a | 0.89 |
| 70226 | Fracking | Blaine County region, Oklahoma | d | d | d | d | c | a | a | d | a | 0.89 |
| 70227 | Fracking | Blaine County region, Oklahoma | d | d | d | d | c | a | a | d | a | 0.89 |
| 70228 | Fracking | Blaine County region, Oklahoma | d | d | d | d | c | a | a | d | a | 0.89 |
| 70229 | Fracking | Blaine County region, Oklahoma | d | d | d | d | c | a | a | d | a | 0.89 |
| 70230 | Fracking | Blaine County region, Oklahoma | d | d | d | d | c | a | a | d | a | 0.89 |
| 70231 | Fracking | Blaine County region, Oklahoma | d | d | d | d | c | a | a | d | a | 0.89 |
| 70232 | Fracking | Blaine County region, Oklahoma | d | d | d | d | c | a | a | d | a | 0.89 |
| 70233 | Fracking | Blaine County region, Oklahoma | d | d | d | d | c | a | a | d | a | 0.89 |
| 70234 | Fracking | Blaine County region, Oklahoma | d | d | d | d | c | a | a | d | a | 0.89 |
| 70235 | Fracking | Blaine County region, Oklahoma | d | d | d | d | c | a | a | d | a | 0.89 |
| 70236 | Fracking | North Canadian County region, Oklahoma | d | d | d | d | c | a | a | a | a | 0.86 |
| 70237 | Fracking | South Canadian County region, Oklahoma | d | d | d | d | c | a | a | a | a | 0.86 |
| 70238 | Fracking | South Canadian County region, Oklahoma | d | d | d | d | c | a | a | a | a | 0.86 |
| 70239 | Fracking | South Canadian County region, Oklahoma | d | d | d | d | c | a | a | a | a | 0.86 |
| 70240 | Fracking | South Canadian County region, Oklahoma | d | d | d | d | c | a | a | a | a | 0.86 |
| 70241 | Fracking | South Canadian County region, Oklahoma | d | d | d | d | c | a | a | a | a | 0.86 |
| 70242 | Fracking | South Canadian County region, Oklahoma | d | d | d | d | c | a | a | a | a | 0.86 |
| 70243 | Fracking | South Canadian County region, Oklahoma | d | d | d | d | c | a | a | a | a | 0.86 |
| 70244 | Fracking | South Canadian County region, Oklahoma | d | d | d | d | c | a | a | a | a | 0.86 |
| 70245 | Fracking | South Canadian County region, Oklahoma | d | d | d | d | c | a | a | a | a | 0.86 |
| 70246 | Fracking | South Canadian County region, Oklahoma | d | d | d | d | c | a | a | a | a | 0.86 |
| 70247 | Fracking | South Canadian County region, Oklahoma | d | d | d | d | c | a | a | a | a | 0.86 |
| 70248 | Fracking | North Grady County region, Oklahoma | d | d | d | d | c | a | a | c | a | 0.86 |
| 70249 | Fracking | North Grady County region, Oklahoma | d | d | d | d | c | a | a | c | a | 0.86 |
| 70250 | Fracking | North Grady County region, Oklahoma | d | d | d | d | c | a | a | c | a | 0.86 |
| 70251 | Fracking | North Grady County region, Oklahoma | d | d | d | d | c | a | a | c | a | 0.86 |
| 70252 | Fracking | North Grady County region, Oklahoma | d | d | d | d | c | a | a | c | a | 0.86 |
| 70253 | Fracking | North Grady County region, Oklahoma | d | d | d | d | c | a | a | c | a | 0.86 |
| 70254 | Fracking | North Grady County region, Oklahoma | d | d | d | d | c | a | a | c | a | 0.86 |
| 70255 | Fracking | South Grady County region, Oklahoma | d | d | d | d | c | a | a | a | a | 0.86 |
| 70256 | Fracking | South Grady County region, Oklahoma | d | d | d | d | c | a | a | a | a | 0.86 |
| 70257 | Fracking | South Grady County region, Oklahoma | d | d | d | d | c | a | a | a | a | 0.86 |
| 70258 | Fracking | South Grady County region, Oklahoma | d | d | d | d | c | a | a | a | a | 0.86 |
| 70259 | Fracking | South Grady County region, Oklahoma | d | d | d | d | c | a | a | a | a | 0.86 |
| 70260 | Fracking | South Grady County region, Oklahoma | d | d | d | d | c | a | a | a | a | 0.86 |
| 70261 | Fracking | South Grady County region, Oklahoma | d | d | d | d | c | a | a | a | a | 0.86 |
| 70262 | Fracking | South Grady County region, Oklahoma | d | d | d | d | c | a | a | a | a | 0.86 |
| 70263 | Fracking | South Grady County region, Oklahoma | d | d | d | d | c | a | a | a | a | 0.86 |
| 70264 | Fracking | South Grady County region, Oklahoma | d | d | d | d | c | a | a | a | a | 0.86 |
| 70265 | Fracking | South Grady County region, Oklahoma | d | d | d | d | c | a | a | a | a | 0.86 |
| 70266 | Fracking | South Grady County region, Oklahoma | d | d | d | d | c | a | a | a | a | 0.86 |
| 70267 | Fracking | South Grady County region, Oklahoma | d | d | d | d | c | a | a | a | a | 0.86 |
| 70268 | Fracking | South Grady County region, Oklahoma | d | d | d | d | c | a | a | a | a | 0.86 |
| 70269 | Fracking | South Grady County region, Oklahoma | d | d | d | d | c | a | a | a | a | 0.86 |
| 70270 | Fracking | South Grady County region, Oklahoma | d | d | d | d | c | a | a | a | a | 0.86 |
| 70271 | Fracking | South Grady County region, Oklahoma | d | d | d | d | c | a | a | a | a | 0.86 |
| 70272 | Fracking | South Grady County region, Oklahoma | d | d | d | d | c | a | a | a | a | 0.86 |
| 70273 | Fracking | South Grady County region, Oklahoma | d | d | d | d | c | a | a | a | a | 0.86 |
| 70274 | Fracking | South Grady County region, Oklahoma | d | d | d | d | c | a | a | a | a | 0.86 |
| 70275 | Fracking | South Grady County region, Oklahoma | d | d | d | d | c | a | a | a | a | 0.86 |
| 70276 | Fracking | South Grady County region, Oklahoma | d | d | d | d | c | a | a | a | a | 0.86 |
| 70277 | Fracking | South Grady County region, Oklahoma | d | d | d | d | c | a | a | a | a | 0.86 |
| 70278 | Fracking | South Grady County region, Oklahoma | d | d | d | d | c | a | a | a | a | 0.86 |
| 70279 | Fracking | South Grady County region, Oklahoma | d | d | d | d | c | a | a | a | a | 0.86 |
| 70280 | Fracking | South Grady County region, Oklahoma | d | d | d | d | c | a | a | a | a | 0.86 |
| 70281 | Fracking | South Grady County region, Oklahoma | d | d | d | d | c | a | a | a | a | 0.86 |
| 70282 | Fracking | South Grady County region, Oklahoma | d | d | d | d | c | a | a | a | a | 0.86 |
| 70283 | Fracking | South Grady County region, Oklahoma | d | d | d | d | c | a | a | a | a | 0.86 |
| 70284 | Fracking | South Grady County region, Oklahoma | d | d | d | d | c | a | a | a | a | 0.86 |
| 70285 | Fracking | South Grady County region, Oklahoma | d | d | d | d | c | a | a | a | a | 0.86 |
| 70286 | Fracking | South Grady County region, Oklahoma | d | d | d | d | c | a | a | a | a | 0.86 |
| 70287 | Fracking | South Grady County region, Oklahoma | d | d | d | d | c | a | a | a | a | 0.86 |
| 70288 | Fracking | Woodward County region, Oklahoma | d | d | d | d | c | a | a | a | a | 0.86 |
| 70289 | Fracking | Woodward County region, Oklahoma | d | d | d | d | c | a | a | a | a | 0.86 |
| 70290 | Fracking | Carter County region, Oklahoma | d | d | d | d | c | a | a | d | a | 0.89 |
| 70291 | Fracking | Carter County region, Oklahoma | d | d | d | d | c | a | a | d | a | 0.89 |
| 70292 | Fracking | Carter County region, Oklahoma | d | d | d | d | c | a | a | d | a | 0.89 |
| 70293 | Fracking | Carter County region, Oklahoma | d | d | d | d | c | a | a | d | a | 0.89 |
| 70294 | Fracking | Carter County region, Oklahoma | d | d | d | d | c | a | a | d | a | 0.89 |
| 70295 | Fracking | Carter County region, Oklahoma | d | d | d | d | c | a | a | d | a | 0.89 |
| 70296 | Fracking | Carter County region, Oklahoma | d | d | d | d | c | a | a | d | a | 0.89 |
| 70297 | Fracking | Carter County region, Oklahoma | d | d | d | d | c | a | a | d | a | 0.89 |
| 70298 | Fracking | Carter County region, Oklahoma | d | d | d | d | c | a | a | d | a | 0.89 |
| 70299 | Fracking | Carter County region, Oklahoma | d | d | d | d | c | a | a | d | a | 0.89 |
| 70300 | Fracking | Carter County region, Oklahoma | d | d | d | d | c | a | a | d | a | 0.89 |
| 70301 | Fracking | Garvin County region, Oklahoma | d | d | d | d | c | a | a | a | a | 0.86 |
| 70302 | Fracking | Garvin County region, Oklahoma | d | d | d | d | c | a | a | a | a | 0.86 |
| 70303 | Fracking | Garvin County region, Oklahoma | d | d | d | d | c | a | a | a | a | 0.86 |
| 70304 | Fracking | Garvin County region, Oklahoma | d | d | d | d | c | a | a | a | a | 0.86 |
| 70305 | Fracking | Garvin County region, Oklahoma | d | d | d | d | c | a | a | a | a | 0.86 |
| 70306 | Fracking | Garvin County region, Oklahoma | d | d | d | d | c | a | a | a | a | 0.86 |
| 70307 | Fracking | Garvin County region, Oklahoma | d | d | d | d | c | a | a | a | a | 0.86 |
| 70308 | Fracking | Garvin County region, Oklahoma | d | d | d | d | c | a | a | a | a | 0.86 |
| 70309 | Fracking | Garvin County region, Oklahoma | d | d | d | d | c | a | a | a | a | 0.86 |
| 70310 | Fracking | Garvin County region, Oklahoma | d | d | d | d | c | a | a | a | a | 0.86 |
| 70311 | Fracking | Garvin County region, Oklahoma | d | d | d | d | c | a | a | a | a | 0.86 |
| 70312 | Fracking | Garvin County region, Oklahoma | d | d | d | d | c | a | a | a | a | 0.86 |
| 70313 | Fracking | Garvin County region, Oklahoma | d | d | d | d | c | a | a | a | a | 0.86 |
| 70314 | Fracking | Marshall County region, Oklahoma | d | d | d | c | c | a | a | a | a | 0.58 |
| 70315 | Fracking | Marshall County region, Oklahoma | d | d | d | c | c | a | a | a | a | 0.58 |
| 70316 | Fracking | Marshall County region, Oklahoma | d | d | d | c | c | a | a | a | a | 0.58 |
| 70317 | Fracking | Marshall County region, Oklahoma | d | d | d | c | c | a | a | a | a | 0.58 |
| 70318 | Fracking | North Stephens County region, Oklahoma | d | d | d | d | c | a | a | a | a | 0.86 |
| 70319 | Fracking | North Stephens County region, Oklahoma | d | d | d | d | c | a | a | a | a | 0.86 |
| 70320 | Fracking | North Stephens County region, Oklahoma | d | d | d | d | c | a | a | a | a | 0.86 |
| 70321 | Fracking | North Stephens County region, Oklahoma | d | d | d | d | c | a | a | a | a | 0.86 |
| 70322 | Fracking | North Stephens County region, Oklahoma | d | d | d | d | c | a | a | a | a | 0.86 |
| 70323 | Fracking | North Stephens County region, Oklahoma | d | d | d | d | c | a | a | a | a | 0.86 |
| 70324 | Fracking | North Stephens County region, Oklahoma | d | d | d | d | c | a | a | a | a | 0.86 |
| 70325 | Fracking | North Stephens County region, Oklahoma | d | d | d | d | c | a | a | a | a | 0.86 |
| 70326 | Fracking | North Stephens County region, Oklahoma | d | d | d | d | c | a | a | a | a | 0.86 |
| 70327 | Fracking | North Stephens County region, Oklahoma | d | d | d | d | c | a | a | a | a | 0.86 |
| 70328 | Fracking | Hughes County, Oklahoma | d | d | d | a | a | a | a | a | a | 0.58 |
| 70329 | Fracking | Hughes County, Oklahoma | d | d | d | a | a | a | a | a | a | 0.58 |
| 70330 | Fracking | Hughes County, Oklahoma | d | d | d | a | a | a | a | a | a | 0.58 |
| 70331 | Fracking | Hughes County, Oklahoma | d | d | d | a | a | a | a | a | a | 0.58 |
| 70332 | Fracking | Hughes County, Oklahoma | d | d | d | a | a | a | a | a | a | 0.58 |
| 70333 | Fracking | Hughes County, Oklahoma | d | d | d | a | a | a | a | a | a | 0.58 |
| 70334 | Fracking | Hughes County, Oklahoma | d | d | d | a | a | a | a | a | a | 0.58 |
| 70335 | Fracking | Hughes County, Oklahoma | d | d | d | a | a | a | a | a | a | 0.58 |
| 70336 | Fracking | Hughes County, Oklahoma | d | d | d | a | a | a | a | a | a | 0.58 |
| 70337 | Fracking | Hughes County, Oklahoma | d | d | d | a | a | a | a | a | a | 0.58 |
| 70338 | Fracking | Hughes County, Oklahoma | d | d | d | a | a | a | a | a | a | 0.58 |
| 70339 | Fracking | Hughes County, Oklahoma | d | d | d | a | a | a | a | a | a | 0.58 |
| 70340 | Fracking | Hughes County, Oklahoma | d | d | d | a | a | a | a | a | a | 0.58 |
| 70341 | Fracking | Hughes County, Oklahoma | d | d | d | a | a | a | a | a | a | 0.58 |
| 70342 | Fracking | Hughes County, Oklahoma | d | d | d | a | a | a | a | a | a | 0.58 |
| 70343 | Fracking | Hughes County, Oklahoma | d | d | d | a | a | a | a | a | a | 0.58 |
| 70344 | Fracking | Hughes County, Oklahoma | d | d | d | a | a | a | a | a | a | 0.58 |
| 70345 | Fracking | Hughes County, Oklahoma | d | d | d | a | a | a | a | a | a | 0.58 |
| 70346 | Fracking | Hughes County, Oklahoma | d | d | d | a | a | a | a | a | a | 0.58 |
| 70347 | Fracking | Hughes County, Oklahoma | d | d | d | a | a | a | a | a | a | 0.58 |
| 70348 | Fracking | Hughes County, Oklahoma | d | d | d | a | a | a | a | a | a | 0.58 |
| 70349 | Fracking | Hughes County, Oklahoma | d | d | d | a | a | a | a | a | a | 0.58 |
| 70350 | Fracking | Hughes County, Oklahoma | d | d | d | a | a | a | a | a | a | 0.58 |
| 70351 | Fracking | Hughes County, Oklahoma | d | d | d | a | a | a | a | a | a | 0.58 |
| 70352 | Fracking | Hughes County, Oklahoma | d | d | d | a | a | a | a | a | a | 0.58 |
| 70353 | Fracking | Hughes County, Oklahoma | d | d | d | a | a | a | a | a | a | 0.58 |
| 70354 | Fracking | Hughes County, Oklahoma | d | d | d | a | a | a | a | a | a | 0.58 |
| 70355 | Fracking | Pittsburgh County region, Oklahoma | d | d | d | d | c | a | a | d | a | 0.89 |
| 70356 | Fracking | Pittsburgh County region, Oklahoma | d | d | d | d | c | a | a | d | a | 0.89 |
| 70357 | Fracking | Pittsburgh County region, Oklahoma | d | d | d | d | c | a | a | d | a | 0.89 |
| 70358 | Fracking | Pittsburgh County region, Oklahoma | d | d | d | d | c | a | a | d | a | 0.89 |
| 70359 | Fracking | Pittsburgh County region, Oklahoma | d | d | d | d | c | a | a | d | a | 0.89 |
| 70360 | Fracking | Pittsburgh County region, Oklahoma | d | d | d | d | c | a | a | d | a | 0.89 |
| 70361 | Fracking | Pittsburgh County region, Oklahoma | d | d | d | d | c | a | a | d | a | 0.89 |
| 70362 | Fracking | Pittsburgh County region, Oklahoma | d | d | d | d | c | a | a | d | a | 0.89 |
| 70363 | Fracking | Pittsburgh County region, Oklahoma | d | d | d | d | c | a | a | d | a | 0.89 |
| 70364 | Fracking | Pittsburgh County region, Oklahoma | d | d | d | d | c | a | a | d | a | 0.89 |
| 70365 | Fracking | Pittsburgh County region, Oklahoma | d | d | d | d | c | a | a | d | a | 0.89 |
| 70366 | Fracking | Pittsburgh County region, Oklahoma | d | d | d | d | c | a | a | d | a | 0.89 |
| 70367 | Fracking | Pittsburgh County region, Oklahoma | d | d | d | d | c | a | a | d | a | 0.89 |
| 70368 | Fracking | Pittsburgh County region, Oklahoma | d | d | d | d | c | a | a | d | a | 0.89 |
| 70369 | Fracking | Pittsburgh County region, Oklahoma | d | d | d | d | c | a | a | d | a | 0.89 |
| 70370 | Fracking | Pittsburgh County region, Oklahoma | d | d | d | d | c | a | a | d | a | 0.89 |
| 70371 | Fracking | Pittsburgh County region, Oklahoma | d | d | d | d | c | a | a | d | a | 0.89 |
| 70372 | Fracking | Pittsburgh County region, Oklahoma | d | d | d | d | c | a | a | d | a | 0.89 |
| 70373 | Fracking | Pittsburgh County region, Oklahoma | d | d | d | d | c | a | a | d | a | 0.89 |
| 70374 | Fracking | Pittsburgh County region, Oklahoma | d | d | d | d | c | a | a | d | a | 0.89 |
| 70375 | Fracking | Pittsburgh County region, Oklahoma | d | d | d | d | c | a | a | d | a | 0.89 |
| 70376 | Fracking | Pittsburgh County region, Oklahoma | d | d | d | d | c | a | a | d | a | 0.89 |
| 70377 | Fracking | Pittsburgh County region, Oklahoma | d | d | d | d | c | a | a | d | a | 0.89 |
| 70378 | Fracking | Pittsburgh County region, Oklahoma | d | d | d | d | c | a | a | d | a | 0.89 |
| 70379 | Fracking | Pittsburgh County region, Oklahoma | d | d | d | d | c | a | a | d | a | 0.89 |
| 70380 | Fracking | Pittsburgh County region, Oklahoma | d | d | d | d | c | a | a | d | a | 0.89 |
| 70381 | Fracking | Pittsburgh County region, Oklahoma | d | d | d | d | c | a | a | d | a | 0.89 |
| 70382 | Fracking | Pittsburgh County region, Oklahoma | d | d | d | d | c | a | a | d | a | 0.89 |
| 70383 | Fracking | Pittsburgh County region, Oklahoma | d | d | d | d | c | a | a | d | a | 0.89 |
| 70384 | Fracking | Pittsburgh County region, Oklahoma | d | d | d | d | c | a | a | d | a | 0.89 |
| 70385 | Fracking | Pittsburgh County region, Oklahoma | d | d | d | d | c | a | a | d | a | 0.89 |
| 70386 | Fracking | Pittsburgh County region, Oklahoma | d | d | d | d | c | a | a | d | a | 0.89 |
| 70387 | Fracking | Pittsburgh County region, Oklahoma | d | d | d | d | c | a | a | d | a | 0.89 |
| 70388 | Fracking | Pittsburgh County region, Oklahoma | d | d | d | d | c | a | a | d | a | 0.89 |
| 70389 | Fracking | Pittsburgh County region, Oklahoma | d | d | d | d | c | a | a | d | a | 0.89 |
| 70390 | Fracking | Pittsburgh County region, Oklahoma | d | d | d | d | c | a | a | d | a | 0.89 |
| 70391 | Fracking | Dawson-Septimus Event Family 1 | d | d | d | d | b | a | c | d | a | 0.86 |
| 70392 | Fracking | Dawson-Septimus Event Family 2 | d | d | d | d | b | a | c | d | a | 0.86 |
| 70393 | Fracking | Dawson-Septimus Event Family 3 | d | d | d | d | b | a | c | d | a | 0.86 |
| 70394 | Fracking | Dawson-Septimus Event Family 4 | d | d | d | d | b | a | c | d | a | 0.86 |
| 70395 | Fracking | Dawson-Septimus Event Family 5 | d | d | d | d | b | a | c | d | a | 0.86 |
| 70396 | Fracking | Dawson-Septimus Event Family 6 | d | d | d | d | b | a | c | d | a | 0.86 |
| 70397 | Fracking | Dawson-Septimus Event Family 7 | d | d | d | d | b | a | c | d | a | 0.86 |
| 70398 | Fracking | Dawson-Septimus Event Family 8 | d | d | d | d | b | a | c | d | a | 0.86 |
| 70399 | Fracking | Dawson-Septimus Event Family 9 | d | d | d | d | b | a | c | d | a | 0.86 |
| 70400 | Fracking | Dawson-Septimus Event Family 10 | d | d | d | d | b | a | c | d | a | 0.86 |
| 70401 | Fracking | Dawson-Septimus Event Family 11 | d | d | d | d | b | a | c | d | a | 0.86 |
| 70402 | Fracking | Dawson-Septimus Event Family 12 | d | d | d | d | b | a | c | d | a | 0.86 |
| 70403 | Fracking | Dawson-Septimus Event Family 13 | d | d | d | d | b | a | c | d | a | 0.86 |
| 70404 | Fracking | Dawson-Septimus Event Family 14 | d | d | d | d | b | a | c | d | a | 0.86 |
| 70405 | Fracking | Dawson-Septimus Event Family 15 | d | d | d | d | b | a | c | d | a | 0.86 |
| 70406 | Fracking | Dawson-Septimus Event Family 16 | d | d | d | d | b | a | c | d | a | 0.86 |
| 70407 | Fracking | Dawson-Septimus Event Family 17 | d | d | d | d | b | a | c | d | a | 0.86 |
| 70408 | Fracking | Dawson-Septimus Event Family 18 | d | d | d | d | b | a | c | d | a | 0.86 |
| 70409 | Fracking | Dawson-Septimus Event Family 19 | d | d | d | d | b | a | c | d | a | 0.86 |
| 70410 | Fracking | Dawson-Septimus Event Family 20 | d | d | d | d | b | a | c | d | a | 0.86 |
| 70411 | Fracking | Dawson-Septimus Event Family 21 | d | d | d | d | b | a | c | d | a | 0.86 |
| 70412 | Fracking | Dawson-Septimus Event Family 22 | d | d | d | d | b | a | c | d | a | 0.86 |
| 80001 | Geothermal | Cerro Prieto (Imperial Valley) | d | d | d | d | b | a | a | c | a | 0.83 |
| 80002 | Geothermal | Laugaland (Holtum) and Kaldárholt | d | d | d | d | b | b | c | a | a | 0.80 |
| 80003 | Geothermal | Pohang (PX-1 and PX-2) | d | d | d | d | b | b | c | c | c | 0.80 |
| 80004 | Geothermal | Salton Sea, California | d | d | d | d | c | c | a | d | a | 0.89 |
| 80005 | Geothermal | The Geysers | d | d | d | d | a | a | a | a | a | 0.86 |
| 80006 | Geothermal | Los Humeros | d | d | d | d | a | a | c | d | d | 0.91 |
| 80007 | Geothermal | Monte Amiata | d | d | d | d | b | a | a | c | a | 0.83 |
| 80008 | Geothermal | Berlín (Well TR8A) | d | d | d | d | b | a | c | d | a | 0.86 |
| 80009 | Geothermal | Hellisheiði (Húsmúli reinjection site) | d | d | d | a | b | b | a | a | a | 0.53 |
| 80010 | Geothermal | Cooper Basin (Habanero 4) | d | d | d | d | a | a | c | d | a | 0.89 |
| 80011 | Geothermal | Cooper Basin (Habanero 1) | d | d | d | d | a | a | a | d | a | 0.89 |
| 80012 | Geothermal | Basel | d | d | d | d | b | b | c | d | a | 0.83 |
| 80013 | Geothermal | Soğukyurt | d | d | d | b | a | a | a | a | a | 0.30 |
| 80014 | Geothermal | Rotokawa | d | d | d | d | a | a | a | a | d | 0.89 |
| 80015 | Geothermal | St. Gallen | d | d | d | d | a | a | a | d | a | 0.89 |
| 80016 | Geothermal | Larderello-Travale | d | a | d | c | b | a | a | c | a | 0.28 |
| 80017 | Geothermal | Mokai | d | d | d | d | b | a | a | a | a | 0.83 |
| 80018 | Geothermal | Svartsengi | d | d | d | d | a | a | a | a | a | 0.86 |
| 80019 | Geothermal | Nesjavellir | d | d | a | a | b | a | a | a | a | 0.28 |
| 80020 | Geothermal | Vendenheim, Robertsau, Strasbourg | x | x | x | x | x | x | x | x | x |  |
| 80021 | Geothermal | Torre Alfina | d | d | d | d | a | a | a | a | a | 0.86 |
| 80022 | Geothermal | Ahuachapan | x | x | x | x | x | x | x | x | x |  |
| 80023 | Geothermal | Reykjanes | d | d | a | c | b | a | a | a | a | 0.28 |
| 80024 | Geothermal | Latera | d | d | d | d | c | a | a | a | a | 0.86 |
| 80025 | Geothermal | Soultz (GPK-3) | d | d | d | d | c | a | a | a | a | 0.86 |
| 80026 | Geothermal | Cooper Basin (Habanero 1 restimulation) | d | d | d | d | a | a | c | a | d | 0.89 |
| 80027 | Geothermal | Coso | d | d | d | d | c | a | c | d | a | 0.89 |
| 80028 | Geothermal | Landau | d | d | d | c | c | a | a | d | a | 0.61 |
| 80029 | Geothermal | Ngatamariki | d | d | d | a | b | a | a | a | a | 0.55 |
| 80030 | Geothermal | Curonian Lagoon | d | d | d | d | c | a | a | a | a | 0.86 |
| 80031 | Geothermal | Olkaria | d | d | d | c | a | a | a | a | a | 0.58 |
| 80032 | Geothermal | Well Paralana 2 | d | d | d | d | b | a | c | a | a | 0.83 |
| 80033 | Geothermal | Puhagan | d | d | d | d | b | a | a | a | a | 0.83 |
| 80034 | Geothermal | Soultz (GPK-2) | d | a | a | a | a | a | a | a | d | 0.06 |
| 80035 | Geothermal | Unterhaching | d | d | a | a | d | a | a | a | a | 0.33 |
| 80036 | Geothermal | Insheim | d | a | a | a | a | a | a | d | a | 0.06 |
| 80037 | Geothermal | Hellisheiði (Well HE-8) | d | d | d | d | b | a | a | a | a | 0.83 |
| 80038 | Geothermal | Newberry | d | d | d | d | b | a | a | a | a | 0.83 |
| 80039 | Geothermal | Pohang (PX-1) | d | a | a | a | a | a | a | a | a | 0.03 |
| 80040 | Geothermal | Krafla | d | d | d | c | c | a | a | d | a | 0.61 |
| 80041 | Geothermal | Çeşneli-Şahyar | d | d | d | b | a | a | a | a | a | 0.30 |
| 80042 | Geothermal | Balmatt (MOL-GT-02) | x | x | x | x | x | x | x | x | x |  |
| 80043 | Geothermal | Alkan-Piyadeler | d | d | d | b | a | a | a | a | a | 0.30 |
| 80044 | Geothermal | Cesano | d | d | a | a | c | a | a | a | a | 0.30 |
| 80045 | Geothermal | Rosemanowes | x | x | x | x | x | x | x | x | x |  |
| 80046 | Geothermal | Ogachi (OGC-1) | d | d | d | a | a | a | a | a | a | 0.58 |
| 80047 | Geothermal | Lahendong | d | d | d | a | a | a | a | d | a | 0.61 |
| 80048 | Geothermal | Hellisheiði(Gráuhnjúkar reinjection site) | d | d | a | d | c | a | a | a | a | 0.58 |
| 80049 | Geothermal | OTN3 well, St1 Geothermal Project, Otaniemi, Espoo | d | a | a | a | a | a | a | d | a | 0.06 |
| 80050 | Geothermal | Los Azufres | a | d | a | a | a | a | a | a | a | 0.28 |
| 80051 | Geothermal | Bad Urach | d | d | a | a | b | a | a | a | a | 0.28 |
| 80052 | Geothermal | Cooper Basin (Habanero 3) | d | a | a | a | a | a | a | a | a | 0.03 |
| 80053 | Geothermal | Desert Peak, Nevada | x | x | x | x | x | x | x | x | x |  |
| 80054 | Geothermal | Cooper Basin (Jolokia 1) | d | d | d | b | a | a | c | a | a | 0.30 |
| 80055 | Geothermal | Rittersshoffen, Alsace | d | d | d | a | a | a | a | a | a | 0.58 |
| 80056 | Geothermal | Örnekköy | d | d | d | b | a | a | a | a | a | 0.30 |
| 80057 | Geothermal | United Downs Deep Geothermal Power Project | d | d | d | a | b | a | a | a | a | 0.55 |
| 80058 | Geothermal | Paralana 2 (Diagnostic Fracture Injection Test) | d | d | a | a | b | a | a | a | a | 0.28 |
| 80059 | Geothermal | Kemaliye | d | d | d | b | a | a | a | a | a | 0.30 |
| 80060 | Geothermal | Fenton Hill, New Mexico | d | d | d | a | a | a | a | d | a | 0.61 |
| 80061 | Geothermal | GeneSys, Hannover | d | a | a | a | a | a | a | a | a | 0.03 |
| 80062 | Geothermal | Fjällbacka | d | d | d | a | b | a | c | a | a | 0.55 |
| 80063 | Geothermal | Hijiori (SKG-2 injection/stimulation) | d | d | d | d | a | a | a | a | a | 0.86 |
| 80064 | Geothermal | Groẞ-Schönebeck | d | a | a | a | b | a | a | a | a | 0.00 |
| 80065 | Geothermal | Hijiori (SkG-2 circulation) | d | d | d | d | a | a | c | d | d | 0.91 |
| 80066 | Geothermal | Baca, New Mexico | a | d | d | d | a | a | a | a | d | 0.86 |
| 80067 | Geothermal | Brady, Nevada | a | d | a | a | a | a | a | a | c | 0.28 |
| 80068 | Geothermal | Raft River, Idaho | x | x | x | x | x | x | x | x | x |  |
| 80069 | Geothermal | Salavatli, Aydin | d | a | a | a | a | a | a | a | a | 0.03 |
| 80070 | Geothermal | Wayang Windu | a | d | a | a | a | a | a | a | a | 0.28 |
| 80071 | Geothermal | Darajat | d | d | d | d | a | a | c | d | a | 0.89 |
| 80072 | Geothermal | Tres Virgenes, LV-06 | d | d | d | d | b | a | a | a | b | 0.80 |
| 80073 | Geothermal | Okuaizu (Yanaizu-Nishiyama) | d | d | d | a | a | a | a | a | a | 0.58 |
| 90001 | Groundwater extraction | Gorkha earthquake, Indo-Gangetic plains | d | d | d | d | b | b | c | d | a | 0.83 |
| 90002 | Groundwater extraction | Iran-Iraq border earthquake | d | d | d | c | a | a | c | d | a | 0.61 |
| 90003 | Groundwater extraction | Lorca | d | d | d | a | c | c | c | d | d | 0.64 |
| 90004 | Groundwater extraction | Lake Kinneret | d | d | d | d | b | a | c | d | d | 0.89 |
| 90005 | Groundwater extraction/Water dam | Jaen (Giribaile reservoir) | d | d | d | d | d | d | c | a | a | 0.91 |
| 90006 | Groundwater (extraction) | Bebedouro, Paraná Basin | d | d | d | d | b | a | a | d | a | 0.86 |
| 90007 | Groundwater extraction | San Joaquin Valley | a | a | a | d | c | a | a | a | d | 0.30 |
| 90008 | Groundwater extraction | Aravalli Delhi fold belt | a | d | d | c | c | a | c | a | d | 0.58 |
| 100001 | Mining | Bachatsky, Kuzbass | d | d | d | a | a | a | a | a | a | 0.58 |
| 100002 | Mining | Changning, Sichuan | d | d | d | d | c | c | c | d | a | 0.89 |
| 100003 | Mining | Volkershausen (Ernst Thaelmann/Merkers mine) | a | d | d | d | a | a | a | a | a | 0.83 |
| 100004 | Mining | Newcastle | d | d | d | d | d | d | a | d | a | 0.94 |
| 100005 | Mining | President Brand Mine, Welkom | d | d | d | a | c | c | c | b | a | 0.61 |
| 100006 | Mining | Moab Khotsong near Orkney | a | d | a | a | a | a | a | b | a | 0.25 |
| 100007 | Mining | Ellalong | a | d | d | d | a | a | a | a | a | 0.83 |
| 100008 | Mining | Maitland | a | d | d | d | a | a | a | a | a | 0.83 |
| 100009 | Mining | Boolaroo | a | d | d | d | a | a | a | a | a | 0.83 |
| 100010 | Mining | Klerksdorp (DRDGold's North West Operations) | d | d | a | a | a | a | a | a | a | 0.30 |
| 100011 | Mining | Attica, New York | x | x | x | x | x | x | x | x | x |  |
| 100012 | Mining | Sunna (Suenna) | x | x | x | x | x | x | x | x | x |  |
| 100013 | Mining | Welkom | d | a | a | a | a | a | a | a | a | 0.03 |
| 100014 | Mining | Solvay mine, Wyoming | d | d | c | a | c | c | c | d | a | 0.33 |
| 100015 | Mining | Morenci Mine, Arizona | d | c | c | c | c | c | a | d | a | 0.06 |
| 100016 | Mining | Umbozero Mine | x | x | x | x | x | x | x | x | x |  |
| 100017 | Mining | Heringen | x | x | x | x | x | x | x | x | x |  |
| 100018 | Mining | Lubin mine | x | x | x | x | x | x | x | x | x |  |
| 100019 | Mining | Hartebeesfontein | x | x | x | x | x | x | x | x | x |  |
| 100020 | Mining | Kalgoorlie Super Pit | a | d | a | a | a | a | a | a | a | 0.28 |
| 100021 | Mining | Wright-Hargreaves mine, Ontario | d | d | a | a | a | a | a | a | a | 0.30 |
| 100022 | Mining | Mosaic Company, Yorkton, Saskatchewan | d | a | a | a | b | a | a | c | a | 0.00 |
| 100023 | Mining | Le Teil Quarry | d | d | d | a | c | a | c | d | d | 0.64 |
| 100024 | Mining | Changning, Sichuan | d | d | a | d | b | a | c | d | a | 0.58 |
| 100025 | Mining | Free State Goldfield | d | d | d | a | c | c | a | d | d | 0.64 |
| 100026 | Mining | Carletonville | d | d | a | a | b | a | a | c | c | 0.28 |
| 100027 | Mining | Solikamsk, Upper Kama | c | a | a | a | a | a | a | a | a | 0.03 |
| 100028 | Mining | Saale (Halle) (Teutschental mine) | x | x | x | x | x | x | x | x | x |  |
| 100029 | Mining | Belchatow | d | d | d | a | c | c | a | c | a | 0.58 |
| 100030 | Mining | Salt mine, Zigong, Sichuan | d | d | a | d | a | a | a | a | a | 0.58 |
| 100031 | Mining | Ibbenbüren | x | x | x | x | x | x | x | x | x |  |
| 100032 | Mining | Dnieper–Donets aulacogen | d | a | a | a | c | c | a | d | a | 0.06 |
| 100033 | Mining | Moss No. 2, Virginia | d | d | a | a | a | a | a | a | a | 0.30 |
| 100034 | Mining | Zhezkazgan Deposit (Zlatoyust-Belovskiy Quarry) | d | d | a | d | a | a | a | a | a | 0.58 |
| 100035 | Mining | Rosvumchorr mine | d | d | a | d | d | d | a | a | a | 0.64 |
| 100036 | Mining | Taeyoung EMC, Geummae-ri, Wonnam-myeon, Uljin-gun, Gyeongsangbuk-do | d | d | d | a | a | a | a | a | a | 0.58 |
| 100037 | Mining | Cacoosing Valley (Sinking Springs), Pennsylvania | c | d | d | b | c | a | c | d | a | 0.30 |
| 100038 | Mining | SKRU-2, Ural Mountains | a | d | d | a | c | a | a | d | a | 0.58 |
| 100039 | Mining | Appin, Tower and West Cliff Collieries | d | d | d | c | a | a | a | a | a | 0.58 |
| 100040 | Mining | Soligorsk (Starobin deposit) | a | d | a | a | a | a | a | d | a | 0.30 |
| 100041 | Mining | Savuka, Carletonville | d | d | d | a | a | a | c | a | a | 0.58 |
| 100042 | Mining | Kachkanar earthquake | x | x | x | x | x | x | x | x | x |  |
| 100043 | Mining | Taiji mine, Beipiao, Liaoning | d | d | d | d | a | a | a | d | a | 0.89 |
| 100044 | Mining | Chayuan mine, Shizhu, Sichuan | x | x | x | x | x | x | x | x | x |  |
| 100045 | Mining | Kurgazakskaya Mine | x | x | x | x | x | x | x | x | x |  |
| 100046 | Mining | Louguanshan #4 well, South Bureau, Sichuan | x | x | x | x | x | x | x | x | x |  |
| 100047 | Mining | Kolyvan Mine, Gorlovsky Coal Basin | d | d | d | d | c | a | c | a | d | 0.89 |
| 100048 | Mining | Taeyoung EMC, Gangwon-do, Samcheok city, Gangwon province | x | x | x | x | x | x | x | x | x |  |
| 100049 | Mining | Weixi mine, Leshan, Sichuan | x | x | x | x | x | x | x | x | x |  |
| 100050 | Mining | Mentougou mine, Beijing, Beijing | d | d | d | a | a | a | a | d | a | 0.61 |
| 100051 | Mining | Willow Creek, Utah | d | d | a | a | a | a | c | a | a | 0.30 |
| 100052 | Mining | Rudna mine | d | d | d | d | c | a | c | d | a | 0.89 |
| 100053 | Mining | Kiruna (Kiirunavaara) mine | d | d | d | c | c | a | c | d | d | 0.64 |
| 100054 | Mining | Reocín mine | x | x | x | x | x | x | x | x | x |  |
| 100055 | Mining | Sangan mine | a | d | a | a | a | a | a | a | a | 0.28 |
| 100056 | Mining | Ruhr area | x | x | x | x | x | x | x | x | x |  |
| 100057 | Mining | Huachu mine, Liuzhi, Guizhou | x | x | x | x | x | x | x | x | x |  |
| 100058 | Mining | Kirovsky Mine, Khibiny Massif (Kola Peninsula) | d | a | a | a | a | a | a | a | a | 0.03 |
| 100059 | Mining | Blinovo-Kamensky Mine | d | a | a | a | a | a | a | a | a | 0.03 |
| 100060 | Mining | Creighton, Ontario | d | d | d | d | a | a | a | a | a | 0.86 |
| 100061 | Mining | Pingyi County, Shandong Province | a | a | d | a | a | a | c | d | a | 0.30 |
| 100062 | Mining | Lorraine | x | x | x | x | x | x | x | x | x |  |
| 100063 | Mining | Buchanan No. 1, Virginia | x | x | x | x | x | x | x | x | x |  |
| 100064 | Mining | Lynch mine, Kentucky | a | d | a | a | c | a | a | d | a | 0.30 |
| 100065 | Mining | Western Deep Levels East | d | d | d | a | a | a | a | a | a | 0.58 |
| 100066 | Mining | Saar (Primsmulde), Saarland | d | d | d | c | b | a | d | a | a | 0.58 |
| 100067 | Mining | Kloof | d | d | a | a | a | a | a | d | a | 0.33 |
| 100068 | Mining | Belle Plaine | x | x | x | x | x | x | x | x | x |  |
| 100069 | Mining | Voerendaal-Kunrade | d | d | d | d | b | a | a | a | d | 0.86 |
| 100070 | Mining | Gran Sasso | d | d | a | d | d | d | c | a | d | 0.66 |
| 100071 | Mining | Crandall Canyon, Utah | d | d | d | d | a | a | d | d | a | 0.91 |
| 100072 | Mining | Deelkraal | x | x | x | x | x | x | x | x | x |  |
| 100073 | Mining | East Driefontein | d | d | a | a | a | a | a | a | a | 0.30 |
| 100074 | Mining | Peissenberg | d | d | d | a | a | a | a | a | a | 0.58 |
| 100075 | Mining | Wacang/Shimacao/Chenjiapo mines, Yichang, Hubei | x | x | x | x | x | x | x | x | x |  |
| 100076 | Mining | King #4, Utah | d | a | d | a | a | a | a | a | a | 0.30 |
| 100077 | Mining | Lynch No. 37, Kentucky | x | x | x | x | x | x | x | x | x |  |
| 100078 | Mining | Wulong mine, Fuxin, Liaoning | d | a | a | a | a | a | a | a | a | 0.03 |
| 100079 | Mining | Bobrek mine | d | d | d | d | a | a | c | d | a | 0.89 |
| 100080 | Mining | Copper Cliff North, Ontario | a | d | a | a | a | a | a | a | a | 0.28 |
| 100081 | Mining | Kidd Creek, Ontario | x | x | x | x | x | x | x | x | x |  |
| 100082 | Mining | Elandsrand | x | x | x | x | x | x | x | x | x |  |
| 100083 | Mining | Pstrowski mine | d | d | a | a | a | a | a | a | a | 0.30 |
| 100084 | Mining | Mechowice mine | d | d | a | a | a | a | a | a | a | 0.30 |
| 100085 | Mining | Szombierki mine | d | d | a | a | a | a | a | a | a | 0.30 |
| 100086 | Mining | CSA Mine, Ostrava-Karvina Coal Basin | d | d | d | d | a | a | a | a | a | 0.86 |
| 100087 | Mining | East Rand Proprietary Mine (ERPM) | a | d | a | a | a | a | a | a | a | 0.28 |
| 100088 | Mining | Nanshan mine, Hegang, Heilongjiang | x | x | x | x | x | x | x | x | x |  |
| 100089 | Mining | Laohutai mine, Fushun, Liaoning | d | d | d | c | a | a | a | a | d | 0.61 |
| 100090 | Mining | Saar/Lorraine | x | x | x | x | x | x | x | x | x |  |
| 100091 | Mining | Mponeng | x | x | x | x | x | x | x | x | x |  |
| 100092 | Mining | Leeudoorn | x | x | x | x | x | x | x | x | x |  |
| 100093 | Mining | Huaibaoshi mine, Zigui, Yichang, Hubei | x | x | x | x | x | x | x | x | x |  |
| 100094 | Mining | Taozhuang mine, Zaozhuang, Shandong | d | d | d | c | a | a | a | a | a | 0.58 |
| 100095 | Mining | Jim Walter Resources, Inc., No. 4, Alabama | x | x | x | x | x | x | x | x | x |  |
| 100096 | Mining | Bingshuijing mine, Yingpan, Liuzhi, Guizhou | x | x | x | x | x | x | x | x | x |  |
| 100097 | Mining | Liu zhi mine, Yingpan, Liuzhi, Guizhou | x | x | x | x | x | x | x | x | x |  |
| 100098 | Mining | Soldier Creek, Utah | x | x | x | x | x | x | x | x | x |  |
| 100099 | Mining | Retsof, New York | x | x | x | x | x | x | x | x | x |  |
| 100100 | Mining | Genesseo, New York | x | x | x | x | x | x | x | x | x |  |
| 100101 | Mining | Tashtagol Mine | x | x | x | x | x | x | x | x | x |  |
| 100102 | Mining | Shunyuan mine, Zaozhuang, Shandong | a | d | a | a | a | a | a | a | a | 0.28 |
| 100103 | Mining | Karnasurt Mine | x | x | x | x | x | x | x | x | x |  |
| 100104 | Mining | Vorkutinskoe earthquake | a | d | d | a | a | a | a | a | a | 0.55 |
| 100105 | Mining | Cottonwood, Utah | d | d | d | a | a | a | a | a | a | 0.58 |
| 100106 | Mining | Saar (Dilsburg Ost), Saarland | d | d | d | d | a | a | c | a | a | 0.86 |
| 100107 | Mining | Mine 15-15bis | x | x | x | x | x | x | x | x | x |  |
| 100108 | Mining | Olympia Dam | a | d | d | a | a | a | a | a | a | 0.55 |
| 100109 | Mining | Lucky Friday Mine, Idaho | d | d | d | a | a | a | a | a | d | 0.61 |
| 100110 | Mining | Stepnyak | a | d | a | a | c | a | a | d | a | 0.30 |
| 100111 | Mining | Olga, West Virginia | c | a | a | a | a | a | a | a | a | 0.00 |
| 100112 | Mining | North Staffordshire (Stoke on Trent) | d | d | d | d | b | a | c | d | a | 0.86 |
| 100113 | Mining | Sunnyside #3, Utah | x | x | x | x | x | x | x | x | x |  |
| 100114 | Mining | Xujiadong 711 mine, Chenzhou, Hunan | x | x | x | x | x | x | x | x | x |  |
| 100115 | Mining | San he jian mine, Xuzhou, Jiangsu | x | x | x | x | x | x | x | x | x |  |
| 100116 | Mining | Cadia | x | x | x | x | x | x | x | x | x |  |
| 100117 | Mining | Zofiowka coal mine, Jastrzebie-Zdroj | x | x | x | x | x | x | x | x | x |  |
| 100118 | Mining | Chengzi mine, Beijing, Beijing | d | a | d | a | a | a | a | a | a | 0.30 |
| 100119 | Mining | Wappingers Falls, New York | d | d | d | a | c | a | c | a | d | 0.61 |
| 100120 | Mining | Cane (Kane) Creek Mine, Utah | d | d | d | d | c | a | c | a | a | 0.86 |
| 100121 | Mining | Trail Mountain, Utah | d | d | d | d | a | a | c | d | a | 0.89 |
| 100122 | Mining | Saar (Primsmulde), Saarland (Roadway construction) | d | d | d | a | a | a | a | a | a | 0.58 |
| 100123 | Mining | Garson, Ontario | x | x | x | x | x | x | x | x | x |  |
| 100124 | Mining | Campbell mine, Ontario | d | d | a | d | a | a | a | a | d | 0.61 |
| 100125 | Mining | Huating mine, Pingliang, Gansu | x | x | x | x | x | x | x | x | x |  |
| 100126 | Mining | Centrum mine | d | d | d | a | a | a | c | a | a | 0.58 |
| 100127 | Mining | Nottinghamshire | d | d | a | a | c | a | a | a | a | 0.30 |
| 100128 | Mining | Niumasi mine, Shaoyang, Hunan | x | x | x | x | x | x | x | x | x |  |
| 100129 | Mining | Mine 14-14bis | x | x | x | x | x | x | x | x | x |  |
| 100130 | Mining | Grängesberg ore mine | x | x | x | x | x | x | x | x | x |  |
| 100131 | Mining | S-Harz | x | x | x | x | x | x | x | x | x |  |
| 100132 | Mining | Star Point #2, Utah | x | x | x | x | x | x | x | x | x |  |
| 100133 | Mining | Xifeng Nan shan mine, Lindong, Guizhou | x | x | x | x | x | x | x | x | x |  |
| 100134 | Mining | Provadia | a | d | a | a | a | a | a | a | a | 0.28 |
| 100135 | Mining | Qixingjiezhen mine, Lianyuan, Hunan | x | x | x | x | x | x | x | x | x |  |
| 100136 | Mining | Yueliangtian mine, Panjiang, Guizhou | x | x | x | x | x | x | x | x | x |  |
| 100137 | Mining | Shanjiaocun mine, Panjiang, Guizhou | x | x | x | x | x | x | x | x | x |  |
| 100138 | Mining | Macassa, Ontario | d | d | d | d | a | a | a | d | a | 0.89 |
| 100139 | Mining | Champion Reef, Kolar Gold field | c | d | d | a | a | a | a | a | c | 0.55 |
| 100140 | Mining | Cory Mine, Saskatchewan | d | d | d | d | a | a | c | a | d | 0.89 |
| 100141 | Mining | Deer Creek, Utah | x | x | x | x | x | x | x | x | x |  |
| 100142 | Mining | Castle Gate #3, Utah | x | x | x | x | x | x | x | x | x |  |
| 100143 | Mining | VP No. 3, Virginia | x | x | x | x | x | x | x | x | x |  |
| 100144 | Mining | Xindong mine, Shaoyang, Hunan | x | x | x | x | x | x | x | x | x |  |
| 100145 | Mining | Skyline #3, Utah | a | d | b | a | a | a | b | a | b | -0.06 |
| 100146 | Mining | Fangshan mine, Beijing, Beijing | d | a | d | d | a | a | a | d | a | 0.61 |
| 100147 | Mining | Mineville, New York | x | x | x | x | x | x | x | x | x |  |
| 100148 | Mining | Polkowice mine | x | x | x | x | x | x | x | x | x |  |
| 100149 | Mining | Quirke mine, Ontario | d | d | d | d | a | a | a | a | a | 0.86 |
| 100150 | Mining | Cleveland, Ohio | d | a | a | a | a | a | a | a | a | 0.03 |
| 100151 | Mining | Galena mine, Idaho | a | d | d | d | a | a | a | a | d | 0.86 |
| 100152 | Mining | En kou mine, Lowde, Hunan | x | x | x | x | x | x | x | x | x |  |
| 100153 | Mining | Dillsburg, Pennsylvania | a | d | d | a | c | a | a | a | a | 0.55 |
| 100154 | Mining | Huayazi mine, Zigui, Yichang, Hubei | x | x | x | x | x | x | x | x | x |  |
| 100155 | Mining | Sheng li mine, Fushun, Liaoning | x | x | x | x | x | x | x | x | x |  |
| 100156 | Mining | Da he bian mine, Shiucheng, Guizhou | x | x | x | x | x | x | x | x | x |  |
| 100157 | Mining | Midlothian | a | d | d | a | a | a | a | a | a | 0.55 |
| 100158 | Mining | Mei tan ba mine, Xifenglun, Hunan | x | x | x | x | x | x | x | x | x |  |
| 100159 | Mining | Niwan mine, Xiangtan, Hunan | x | x | x | x | x | x | x | x | x |  |
| 100160 | Mining | Benxi Caitun mine, Shenyang, Liaoning | x | x | x | x | x | x | x | x | x |  |
| 100161 | Mining | Baidong mine, Datong, Shanxi | x | x | x | x | x | x | x | x | x |  |
| 100162 | Mining | Sijiaotian mine, Yingpan, Liuzhi, Guizhou | x | x | x | x | x | x | x | x | x |  |
| 100163 | Mining | Dizong mine, Yingpan, Liuzhi, Guizhou | x | x | x | x | x | x | x | x | x |  |
| 100164 | Mining | Dayong mine, Yingpan, Liuzhi, Guizhou | x | x | x | x | x | x | x | x | x |  |
| 100165 | Mining | Strathcona, Ontario | a | d | d | a | a | a | c | a | a | 0.55 |
| 100166 | Mining | Dahuatang mine, Shaoyang, Hunan | x | x | x | x | x | x | x | x | x |  |
| 100167 | Mining | Qingshan mine, Lianyuan, Hunan | x | x | x | x | x | x | x | x | x |  |
| 100168 | Mining | San Nicolás (Mieres) and Montsacro (Morcín – Riosa) mines | a | d | a | a | c | a | a | d | a | 0.30 |
| 100169 | Mining | Zingruvan | a | d | a | d | a | a | a | a | a | 0.55 |
| 100170 | Mining | Bingham Canyon Mine, Utah | d | d | d | d | c | a | a | a | a | 0.86 |
| 100171 | Mining | Longfeng mine, Fushun, Liaoning | d | d | d | a | a | a | a | a | d | 0.61 |
| 100172 | Mining | Doulishan mine, Lowde, Hunan | x | x | x | x | x | x | x | x | x |  |
| 100173 | Mining | Yan guan mine, Zigui, Yichang, Hubei | x | x | x | x | x | x | x | x | x |  |
| 100174 | Mining | Gardanne | c | d | a | d | a | a | a | a | a | 0.55 |
| 100175 | Mining | Lo Tacón (Torre Pacheco) | c | d | d | d | a | a | a | a | a | 0.83 |
| 100176 | Mining | MFS Faido (Gotthard basetunnel) | d | d | d | d | a | a | c | a | a | 0.86 |
| 100177 | Mining | Fraser, Ontario | x | x | x | x | x | x | x | x | x |  |
| 100178 | Mining | Dogye | d | d | a | a | a | a | a | d | a | 0.33 |
| 100179 | Mining | Lompoc diatomite mine, California | d | a | d | a | a | a | c | a | a | 0.30 |
| 100180 | Mining | Florida, New York | x | x | x | x | x | x | x | x | x |  |
| 100181 | Mining | Gangdong mine, Shuangyashan, Heilongjiang | x | x | x | x | x | x | x | x | x |  |
| 100182 | Mining | Dannemora | d | d | d | d | a | a | a | a | a | 0.86 |
| 100183 | Mining | Qiao tou he mine, Lowde, Hunan | x | x | x | x | x | x | x | x | x |  |
| 100184 | Mining | Rotherham (Yorkshire) | x | x | x | x | x | x | x | x | x |  |
| 100185 | Mining | Kaiyang mine, Jinzhong, Kaiyang, Guizhou | x | x | x | x | x | x | x | x | x |  |
| 100186 | Mining | Bargoed Mid Glamorgan (South Wales) | x | x | x | x | x | x | x | x | x |  |
| 100187 | Mining | TauTona, Carletonville | a | d | d | a | a | a | a | a | a | 0.55 |
| 100188 | Mining | Craig, Ontario | x | x | x | x | x | x | x | x | x |  |
| 100189 | Mining | Wujek mine | x | x | x | x | x | x | x | x | x |  |
| 100190 | Mining | Ziemowit mine | x | x | x | x | x | x | x | x | x |  |
| 100191 | Mining | Sunagawa mine | d | d | d | d | a | a | c | d | a | 0.89 |
| 100192 | Mining | Buxton (Derbyshire) | d | d | a | d | a | a | a | a | a | 0.58 |
| 100193 | Mining | Jinhuagong mine, Datong, Shanxi | x | x | x | x | x | x | x | x | x |  |
| 100194 | Mining | Sunderland (Durham and Northumberland) | a | d | a | d | a | a | a | a | a | 0.55 |
| 100195 | Mining | Montfort | x | x | x | x | x | x | x | x | x |  |
| 100196 | Mining | Mayrau mine | d | d | d | d | a | a | a | c | a | 0.86 |
| 100197 | Mining | Shuikoushan mine, Hengnan, Hunan | x | x | x | x | x | x | x | x | x |  |
| 100198 | Mining | Bolton (Lancashire) | a | d | d | a | a | a | a | a | a | 0.55 |
| 100199 | Mining | Shi xia jiang mine, Shaoyang, Hunan | x | x | x | x | x | x | x | x | x |  |
| 100200 | Mining | Oxy Geismar Cavern, Napoleon salt dome, Assumption Parish, Louisiana | a | d | d | a | a | a | c | a | a | 0.55 |
| 100201 | Mining | Boulby mine, North Yorkshire | a | d | a | a | c | a | a | a | a | 0.28 |
| 100202 | Mining | Pyhäsalmi | d | d | d | d | a | a | a | d | a | 0.89 |
| 100203 | Mining | Beatrice, Virginia | d | d | d | a | a | a | a | a | a | 0.58 |
| 100204 | Mining | Dale, New York | d | a | a | a | a | a | a | a | a | 0.03 |
| 100205 | Mining | Moonee Colliery | x | x | x | x | x | x | x | x | x |  |
| 100206 | Mining | Springfield Pike Quarry, Pennsylvania | x | x | x | x | x | x | x | x | x |  |
| 100207 | Mining | Field II, Ocnele Mari | d | d | d | d | a | a | c | d | d | 0.91 |
| 100208 | Mining | Dagandsham Hydropower Station | d | d | d | d | a | a | a | a | d | 0.89 |
| 100209 | Mining | Arkema-Vauvert | d | d | d | a | a | a | c | a | a | 0.58 |
| 100210 | Mining | Underground Research Laboratory, Manitoba | d | d | d | a | a | a | c | a | d | 0.61 |
| 100211 | Mining | Ophirton | x | x | x | x | x | x | x | x | x |  |
| 100212 | Mining | Wilkes-Barre, Pennsylvania | a | a | a | a | c | a | a | a | a |  |
| 100213 | Mining | Raibl mine, Cave del Predil | x | x | x | x | x | x | x | x | x |  |
| 100214 | Mining | Dongguashan (Shizishan copper mine), Tongling, Hunan (Roadway construction) | a | d | d | a | a | a | a | a | a | 0.55 |
| 100215 | Mining | Tianshengqiao II Hydropower Station (Head race tunnel construction) | d | d | d | a | a | a | a | a | a | 0.58 |
| 100216 | Mining | Brunswick No. 12 mine | d | d | d | a | a | a | a | d | a | 0.61 |
| 100217 | Mining | Queenstown, Tasmania | x | x | x | x | x | x | x | x | x |  |
| 100218 | Mining | Chinakuri Colliery | a | a | a | a | a | a | a | a | d | 0.03 |
| 100219 | Mining | Gluboky Mine, Streltsovsk | a | d | d | a | a | a | a | a | a | 0.55 |
| 100220 | Mining | Denison mine, Ontario | a | d | d | a | a | a | a | a | a | 0.55 |
| 100221 | Mining | Miike mine | x | x | x | x | x | x | x | x | x |  |
| 100222 | Mining | Mount Charlotte Mine | x | x | x | x | x | x | x | x | x |  |
| 100223 | Mining | Fuli mine, Hegang, Heilongjiang | x | x | x | x | x | x | x | x | x |  |
| 100224 | Mining | Didao mine, Jixi, Heilongjiang | x | x | x | x | x | x | x | x | x |  |
| 100225 | Mining | Tiechang mine, Tonghua, Jilin | x | x | x | x | x | x | x | x | x |  |
| 100226 | Mining | Hongtoushan mine, Fushun, Liaoning | x | x | x | x | x | x | x | x | x |  |
| 100227 | Mining | Gaode mine, Fuxin, Liaoning | a | a | a | d | c | a | a | a | c | 0.28 |
| 100228 | Mining | Dongliang mine, Fuxin, Liaoning | x | x | x | x | x | x | x | x | x |  |
| 100229 | Mining | Guanshan mine, Beipiao, Liaoning | x | x | x | x | x | x | x | x | x |  |
| 100230 | Mining | Datai mine, Beijing, Beijing | x | x | x | x | x | x | x | x | x |  |
| 100231 | Mining | Tongjialiang mine, Datong, Shanxi | x | x | x | x | x | x | x | x | x |  |
| 100232 | Mining | Meiyukou mine, Datong, Shanxi | x | x | x | x | x | x | x | x | x |  |
| 100233 | Mining | Yongdingzhuang mine, Datong, Shanxi | x | x | x | x | x | x | x | x | x |  |
| 100234 | Mining | Bayi mine, Zaozhuang, Shandong | x | x | x | x | x | x | x | x | x |  |
| 100235 | Mining | Chaili mine, Zaozhuang, Shandong | x | x | x | x | x | x | x | x | x |  |
| 100236 | Mining | Wumei mine, Hebi, Henan | x | x | x | x | x | x | x | x | x |  |
| 100237 | Mining | Shier (Shi'er kuang?) mine, Pingdingshan, Henan | x | x | x | x | x | x | x | x | x |  |
| 100238 | Mining | Quantai mine, Xuzhou, Jiangsu | a | a | d | a | a | a | a | a | a | 0.28 |
| 100239 | Mining | Qishan mine, Xuzhou, Jiangsu | x | x | x | x | x | x | x | x | x |  |
| 100240 | Mining | Zhangxiaolou mine, Xuzhou, Jiangsu | x | x | x | x | x | x | x | x | x |  |
| 100241 | Mining | Zhangji mine, Xuzhou, Jiangsu | x | x | x | x | x | x | x | x | x |  |
| 100242 | Mining | Leigu mine, Mianyang Beichuan, Sichuan | x | x | x | x | x | x | x | x | x |  |
| 100243 | Mining | Tianchi mine, Mianzhu, Sichuan | x | x | x | x | x | x | x | x | x |  |
| 100244 | Mining | Yanshitai mine, Wansheng district, Nantong, Chongqing | x | x | x | x | x | x | x | x | x |  |
| 100245 | Mining | Bajing mine, Gaoan, Jiangxi | x | x | x | x | x | x | x | x | x |  |
| 100246 | Mining | Tungsten ore mine, Jiangxi | x | x | x | x | x | x | x | x | x |  |
| 100247 | Mining | Manganese mine, Zunyi, Guizhou | x | x | x | x | x | x | x | x | x |  |
| 100248 | Mining | South manganese mine, Huayuan, Hunan | x | x | x | x | x | x | x | x | x |  |
| 100249 | Mining | Manganese mine, Taojiang, Hunan | x | x | x | x | x | x | x | x | x |  |
| 100250 | Mining | Phosphorus mine, Yichang, Hubei | x | x | x | x | x | x | x | x | x |  |
| 100251 | Mining | Yangmuxi mine, Changyang, Yichang, Hubei | x | x | x | x | x | x | x | x | x |  |
| 100252 | Mining | Songyi mine, Yichang, Hubei | x | x | x | x | x | x | x | x | x |  |
| 100253 | Mining | Gaofeng mine, Dachangjingtian, Guangxi | x | x | x | x | x | x | x | x | x |  |
| 100254 | Mining | Tongkeng mine, Dachangjingtian, Guangxi | x | x | x | x | x | x | x | x | x |  |
| 100255 | Mining | Manganese mine, Dounan, Yunnan | x | x | x | x | x | x | x | x | x |  |
| 100256 | Mining | Manganese mine, Heqing, Yunnan | x | x | x | x | x | x | x | x | x |  |
| 100257 | Mining | Dongguashan (Shizishan copper mine), Tongling, Hunan | x | x | x | x | x | x | x | x | x |  |
| 100258 | Mining | Zhenxing mine, Hegang, Heilongjiang | x | x | x | x | x | x | x | x | x |  |
| 100259 | Mining | Yingcheng mine, Shulang, Jilin | x | x | x | x | x | x | x | x | x |  |
| 100260 | Mining | Xian mine, Liaoyuan, Jilin | x | x | x | x | x | x | x | x | x |  |
| 100261 | Mining | Tai xin mine, Liaoyuan, Jilin | x | x | x | x | x | x | x | x | x |  |
| 100262 | Mining | Benxi Niu xin tai mine, Shenyang, Liaoning | x | x | x | x | x | x | x | x | x |  |
| 100263 | Mining | Binggou mine, Jianchang county, Liaoning | x | x | x | x | x | x | x | x | x |  |
| 100264 | Mining | Chang/Zhang gou yu mine, Beijing, Beijing | x | x | x | x | x | x | x | x | x |  |
| 100265 | Mining | Muchengjian mine, Beijing, Beijing | x | x | x | x | x | x | x | x | x |  |
| 100266 | Mining | Tang shan mine, Kailuan, Hebei | x | x | x | x | x | x | x | x | x |  |
| 100267 | Mining | Guan tai mine, Cixian, Hebei | x | x | x | x | x | x | x | x | x |  |
| 100268 | Mining | Xin zhou yao mine, Datong, Shanxi | d | a | c | d | a | a | a | a | a | 0.30 |
| 100269 | Mining | Huafeng mine, Xinwen, Shandong | x | x | x | x | x | x | x | x | x |  |
| 100270 | Mining | Sun cun mine, Xinwen, Shandong | a | d | d | a | a | a | a | a | a | 0.55 |
| 100271 | Mining | Zhangzhuan mine, Xinwen, Shandong | x | x | x | x | x | x | x | x | x |  |
| 100272 | Mining | Pan xi mine, Xinwen, Shandong | x | x | x | x | x | x | x | x | x |  |
| 100273 | Mining | Dong tan mine, Yankuang, Shandong | x | x | x | x | x | x | x | x | x |  |
| 100274 | Mining | Bao dian mine, Yankuang, Shandong | x | x | x | x | x | x | x | x | x |  |
| 100275 | Mining | #2 mine, Weishanhu, Shandong | x | x | x | x | x | x | x | x | x |  |
| 100276 | Mining | Qianqiu mine, Yima, Henan | x | x | x | x | x | x | x | x | x |  |
| 100277 | Mining | Yaoqiao mine, Datun, Jiangsu | x | x | x | x | x | x | x | x | x |  |
| 100278 | Mining | Kong zhuang mine, Datun, Jiangsu | x | x | x | x | x | x | x | x | x |  |
| 100279 | Mining | Wuyi mine, Shanxi | x | x | x | x | x | x | x | x | x |  |
| 100280 | Mining | Nantong mine, Nantong, Chongqing | x | x | x | x | x | x | x | x | x |  |
| 100281 | Mining | Hua gu shan mine, Xinyu, Jiangxi | x | x | x | x | x | x | x | x | x |  |
| 100282 | Mining | Fengdouyan, Jiupanshan, Qishuping and Beitou mines, Jiupanshan, Yichang, Hubei | x | x | x | x | x | x | x | x | x |  |
| 100283 | Mining | Berezniki-1 Mine | d | d | d | a | a | a | a | d | d | 0.64 |
| 100284 | Mining | Southern Colliery, German Creek | a | a | a | d | c | a | a | a | a | 0.28 |
| 100285 | Mining | El Teniente | a | d | d | a | c | a | d | a | a | 0.58 |
| 100286 | Mining | Horonai | a | d | d | d | a | a | a | a | d | 0.86 |
| 100287 | Mining | Road tunnel | a | d | a | a | a | a | a | a | a | 0.28 |
| 100288 | Mining | Head race tunnel | d | d | d | a | a | a | a | a | a | 0.58 |
| 100289 | Mining | Simplon Tunnel | d | a | a | a | a | a | a | a | a | 0.03 |
| 100290 | Mining | Shimizu Tunnel | d | a | a | a | a | a | a | a | a | 0.03 |
| 100291 | Mining | Kanetsu (Kan-Etsu) Tunnel | d | d | d | a | a | a | a | a | a | 0.58 |
| 100292 | Mining | Forsmark Nuclear Plant (Hydraulic tunnels construction) | x | x | x | x | x | x | x | x | x |  |
| 100293 | Mining | Ritsem Traffic Tunnel | x | x | x | x | x | x | x | x | x |  |
| 100294 | Mining | Mishraq | d | d | a | d | a | a | a | a | a | 0.58 |
| 100295 | Mining | Yuzixi I Hydropower Station (Head race tunnel construction) | d | d | a | a | a | a | a | a | a | 0.30 |
| 100296 | Mining | Erlangshan Tunnel (Sichuan-Tibet Highway) | a | d | d | a | a | a | a | a | a | 0.55 |
| 100297 | Mining | Qinling Railway Tunnel | d | a | d | a | a | a | a | a | a | 0.30 |
| 100298 | Mining | Cangling Tunnel (Taizhou-Jiyun Highway) | d | d | d | a | a | a | a | a | a | 0.58 |
| 100299 | Mining | Pubugou Hydropower Station | d | d | d | a | a | a | a | a | a | 0.58 |
| 100300 | Mining | Jinping II Hydropower Station (Auxiliary tunnel) | a | d | d | a | a | a | a | a | a | 0.55 |
| 100301 | Mining | Lujialiang Tunnel (Chongqing-Yichang Highway) | d | d | d | a | a | a | a | a | a | 0.58 |
| 100302 | Mining | Malmberget | d | d | d | a | a | a | a | a | a | 0.58 |
| 100303 | Mining | Ridgeway Deep block cave mine | d | a | a | a | a | a | c | a | a | 0.03 |
| 110001 | Nuclear explosions | Cannikin | d | d | d | d | d | d | d | d | d | 1.00 |
| 110002 | Nuclear explosions | Novaya Zemlya site | d | d | d | a | d | d | a | a | a | 0.64 |
| 110003 | Nuclear explosions | Atrisco, Nevada | x | x | x | x | x | x | x | x | x |  |
| 110004 | Nuclear explosions | Milrow | d | d | d | d | c | a | c | d | d | 0.91 |
| 110005 | Nuclear explosions | Benham, Nevada | d | d | d | c | b | a | c | d | a | 0.58 |
| 110006 | Nuclear explosions | 6th nuclear test (DPRK-6) | d | d | d | a | d | d | c | d | a | 0.66 |
| 110007 | Nuclear explosions | Jorum, Nevada | d | d | d | c | b | a | c | d | a | 0.58 |
| 110008 | Nuclear explosions | 3rd nuclear test (DPRK-3) | d | d | a | a | d | a | a | a | a | 0.33 |
| 110009 | Nuclear explosions | 4th nuclear test (DPRK-4) | d | d | a | a | b | a | a | a | a | 0.28 |
| 110010 | Nuclear explosions | Purse, Nevada | d | d | d | c | b | a | a | d | a | 0.58 |
| 110011 | Nuclear explosions | Handley, Nevada | d | d | d | b | b | a | c | d | a | 0.30 |
| 110012 | Nuclear explosions | 5th nuclear test (DPRK-5) | d | d | a | a | b | a | a | a | a | 0.28 |
| 110013 | Nuclear explosions | Faultless | a | d | a | d | c | a | a | a | a | 0.55 |
| 110014 | Nuclear explosions | Hard Hat, Nevada | d | b | a | a | c | a | a | d | a | -0.22 |
| 110015 | Nuclear explosions | Rex, Nevada | x | x | x | x | x | x | x | x | x |  |
| 110016 | Nuclear explosions | Halfbeak, Nevada | x | x | x | x | x | x | x | x | x |  |
| 110017 | Nuclear explosions | Greeley, Nevada | x | x | x | x | x | x | x | x | x |  |
| 110018 | Nuclear explosions | Bourbon, Nevada | x | x | x | x | x | x | x | x | x |  |
| 110019 | Nuclear explosions | Buff, Nevada | x | x | x | x | x | x | x | x | x |  |
| 110020 | Nuclear explosions | Charcoal, Nevada | x | x | x | x | x | x | x | x | x |  |
| 110021 | Nuclear explosions | Chartreuse | x | x | x | x | x | x | x | x | x |  |
| 110022 | Nuclear explosions | Nash, Nevada | x | x | x | x | x | x | x | x | x |  |
| 110023 | Nuclear explosions | Dumont, Nevada | x | x | x | x | x | x | x | x | x |  |
| 110024 | Nuclear explosions | Tan, Nevada | x | x | x | x | x | x | x | x | x |  |
| 110025 | Nuclear explosions | Boxcar, Nevada | d | a | a | a | b | a | a | a | a | 0.00 |
| 110026 | Nuclear explosions | Duryea, Nevada | x | x | x | x | x | x | x | x | x |  |
| 110027 | Nuclear explosions | Scotch, Nevada | x | x | x | x | x | x | x | x | x |  |
| 110028 | Nuclear explosions | Degelen site of Semipalatinsk Test Site | d | d | a | a | c | a | a | a | a | 0.30 |
| 120001 | Oil and Gas | Fashing Region (D Cluster) | d | d | a | d | a | a | a | a | a | 0.58 |
| 120002 | Oil and Gas | Dimmit County (K Cluster), Texas | d | d | d | d | d | d | a | d | a | 0.94 |
| 120003 | Oil and Gas | Dimmit County (M Cluster), Texas | d | d | a | d | d | d | a | d | a | 0.66 |
| 120004 | Oil and Gas | Fashing Region (H Cluster) | d | d | a | d | c | a | a | d | a | 0.61 |
| 120005 | Oil and Gas | Fashing Region (G Cluster) | d | d | a | d | c | a | a | d | a | 0.61 |
| 120006 | Oil and Gas | Fashing Region (C Cluster) | d | d | a | d | c | a | a | d | a | 0.61 |
| 120007 | Oil and Gas | Fashing Region (Event B) | d | d | a | d | c | a | a | a | a | 0.58 |
| 120008 | Oil and Gas | Dimmit County (L Cluster), Texas | d | d | a | d | d | d | a | d | a | 0.66 |
| 130001 | Oil and Gas/Waste fluid injection | Cavone and San Giacomo fields, Mirandola License (Emilia sequence) | d | d | d | d | c | a | c | d | a | 0.89 |
| 130002 | Oil and Gas/Waste fluid injection | Fashing Region (F Cluster), Texas | d | d | d | c | b | a | a | d | a | 0.58 |
| 130003 | Oil and Gas/Waste fluid injection | Bakken, North Dakota | d | d | d | d | c | a | a | a | a | 0.86 |
| 130004 | Oil and Gas/Waste fluid injection | Cedar Creek Anticline, Montana | d | d | d | d | c | a | a | a | a | 0.86 |
| 140001 | Research | Rangely, Colorado | d | d | d | d | a | a | c | a | a | 0.86 |
| 140002 | Research | Wairakei | d | d | d | d | a | a | c | b | d | 0.86 |
| 140003 | Research | Tongonan Geothermal field | d | d | d | d | d | d | c | d | a | 0.94 |
| 140004 | Research | Matsushiro | d | d | d | d | b | b | c | d | a | 0.83 |
| 140005 | Research | KTB | d | d | d | d | c | c | c | d | a | 0.89 |
| 140006 | Research | WFSD-3P | d | d | d | d | a | a | a | a | a | 0.86 |
| 140007 | Research | KTB | d | d | d | d | a | a | a | d | a | 0.89 |
| 140008 | Research | Nojima | d | d | d | d | b | a | a | d | d | 0.89 |
| 140009 | Research | KTB | d | d | d | a | b | a | a | a | a | 0.55 |
| 140010 | Research | Cerville-Buissoncourt | d | d | d | b | d | d | a | d | a | 0.39 |
| 140011 | Research | Laboratoire Souterrain à Bas Bruit | d | d | d | c | a | a | a | d | d | 0.64 |
| 140012 | Research | Hope mine | d | d | d | d | b | d | a | a | a | 0.86 |
| 140013 | Research | Frio Formation, Beaumont, near Jasper County, Texas | d | d | d | a | a | a | a | a | a | 0.58 |
| 140014 | Research | SB3, Deep Underground rock Laboratory (DUG Lab) at Grimsel Test Site (GTS) | d | d | d | d | a | a | a | a | a | 0.86 |
| 150001 | Waste fluid disposal | Pawnee, Oklahoma | d | d | d | a | c | c | c | c | d | 0.61 |
| 150002 | Waste fluid disposal | Prague, Oklahoma | d | d | d | a | a | a | c | c | a | 0.58 |
| 150003 | Waste fluid disposal | Dawson Creek, British Columbia | d | d | b | a | a | a | a | c | a | 0.03 |
| 150004 | Waste fluid disposal | Raton Basin, Colorado and New Mexico | d | d | d | d | b | a | c | d | a | 0.86 |
| 150005 | Waste fluid disposal | Rongchang gas field | d | d | d | d | c | c | c | d | a | 0.89 |
| 150006 | Waste fluid disposal | Fairview, Oklahoma | d | d | d | d | a | a | c | d | a | 0.89 |
| 150007 | Waste fluid disposal | Cushing, Oklahoma | d | d | d | d | d | d | c | d | d | 0.97 |
| 150008 | Waste fluid disposal | Mentone, Texas | d | d | d | d | a | a | a | d | a | 0.89 |
| 150009 | Waste fluid disposal | Painesville (Perry), Ohio | d | d | d | d | c | a | a | c | a | 0.86 |
| 150010 | Waste fluid disposal | Milan, Summer County, Kansas | d | d | d | d | d | d | c | d | d | 0.97 |
| 150011 | Waste fluid disposal | Rocky Mountain Arsenal (Denver), Colorado | d | d | d | d | d | d | a | a | d | 0.94 |
| 150012 | Waste fluid disposal | Rubiales oil field (Puerto Gaitán sequence), Puerto Gaitán | d | d | d | d | b | b | a | d | a | 0.83 |
| 150013 | Waste fluid disposal | Timpson, East Texas | d | d | d | c | c | a | c | a | c | 0.58 |
| 150014 | Waste fluid disposal | Arkansas | d | d | d | d | b | a | c | d | a | 0.86 |
| 150015 | Waste fluid disposal | Kern River, Kern County, California | d | d | a | d | c | a | a | d | a | 0.61 |
| 150016 | Waste fluid disposal | Tejon, Central Valley (WWF), Kern County, California | d | d | d | d | b | b | a | d | a | 0.83 |
| 150017 | Waste fluid disposal | Huangjiachang gas field | d | d | d | d | c | a | c | d | a | 0.89 |
| 150018 | Waste fluid disposal | Paradox Valley, Colorado | d | d | d | d | c | a | c | a | a | 0.86 |
| 150019 | Waste fluid disposal | Ashtabula, Ohio | d | d | d | a | c | a | c | d | d | 0.64 |
| 150020 | Waste fluid disposal | Lost Hills, Kern County, California | d | d | a | d | b | a | a | a | a | 0.55 |
| 150021 | Waste fluid disposal | Dagger Draw, New Mexico | d | d | d | a | b | a | a | d | a | 0.58 |
| 150022 | Waste fluid disposal | Harper County, Kansas | d | d | d | d | c | a | c | a | a | 0.86 |
| 150023 | Waste fluid disposal | Marcotte oil field (Palco), Kansas | a | d | a | a | b | a | a | a | a | 0.25 |
| 150024 | Waste fluid disposal | Cordel (Brazeau Cluster) | d | d | d | d | b | a | a | d | d | 0.89 |
| 150025 | Waste fluid disposal | Jones, Oklahoma | d | c | d | d | b | a | c | d | d | 0.61 |
| 150026 | Waste fluid disposal | Graham (Montney Trend) | d | d | a | a | b | a | a | a | a | 0.28 |
| 150027 | Waste fluid disposal | Guthrie, Oklahoma | a | d | d | a | c | a | c | d | a | 0.58 |
| 150028 | Waste fluid disposal | Venus, Texas | d | d | d | d | d | d | c | d | d | 0.97 |
| 150029 | Waste fluid disposal | Musreau Lake, Alberta | d | d | d | d | c | a | c | a | a | 0.86 |
| 150030 | Waste fluid disposal | Youngstown, Ohio | d | d | d | d | d | d | c | d | a | 0.94 |
| 150031 | Waste fluid disposal | Lake Charles, Louisiana | x | x | x | x | x | x | x | x | x |  |
| 150032 | Waste fluid disposal | Cleburne, Texas | d | d | d | a | d | d | c | a | a | 0.64 |
| 150033 | Waste fluid disposal | Braxton County, West Virginia | d | d | a | a | c | a | c | c |  | 0.30 |
| 150034 | Waste fluid disposal | Dallas-Fort Worth, Texas | d | d | d | d | d | d | a | a | a | 0.91 |
| 150035 | Waste fluid disposal | Greeley, Colorado | d | d | a | a | d | a | a | a | a | 0.33 |
| 150036 | Waste fluid disposal | Long Run-1 well, Washington County, Ohio | d | d | d | d | d | d | a | d | d | 0.97 |
| 150037 | Waste fluid disposal | Pintail (Montney Trend) | d | d | a | d | a | a | a | a | a | 0.58 |
| 150038 | Waste fluid disposal | Anadarko Basin (Cluster A) | d | d | a | d | a | a | a | a | a | 0.58 |
| 150039 | Waste fluid disposal | El Dorado, Arkansas | d | a | a | a | a | a | a | a | a | 0.03 |
| 150040 | Waste fluid disposal | Lillian (J-A cluster), Barnett Shale, Texas | d | d | a | a | d | d | a | a | a | 0.36 |
| 150041 | Waste fluid disposal | Avoca, New York | a | a | a | a | c | a | a | a | a | 0.00 |
| 150042 | Waste fluid disposal | Oklahoma-Texas Border (Cluster B) | d | d | a | d | b | a | a | a | a | 0.55 |
| 150043 | Waste fluid disposal | Trumbull County, Ohio | d | d | d | d | c | a | a | d | d | 0.91 |
| 150044 | Waste fluid disposal | Val d'Agri oil field (CM2 well) | d | d | d | d | c | a | c | d | a | 0.89 |
| 150045 | Waste fluid disposal | Fashing Region (A Cluster), Texas | d | d | a | d | c | a | a | a | a | 0.58 |
| 150046 | Waste fluid disposal | Oklahoma Panhandle (Cluster E) | d | d | a | b | b | a | a | c | a | 0.00 |
| 150047 | Waste fluid disposal | Dimmit County (Event J), Texas | d | d | a | b | c | a | a | a | a | 0.03 |
| 150048 | Waste fluid disposal | Center, Texas | b |  |  |  |  |  |  |  |  | -1.00 |
| 150049 | Waste fluid disposal | Cedar Creek Anticline, North Dakota | d | d | a | d | c | a | a | a | a | 0.58 |
| 160001 | Water reservoir impoundment | Zipingpu (Wenchuan earthquake) | d | d | d | a | a | a | a | d | a | 0.61 |
| 160002 | water reservoir impoundment | Lake Hebgen , Montana | a | d | a | a | a | a | a | a | a | 0.28 |
| 160003 | Water reservoir impoundment | Polyphyto | d | d | d | d | b | b | a | d | a | 0.83 |
| 160004 | Water reservoir impoundment | Koyna | d | d | d | d | a | a | c | d | a | 0.89 |
| 160005 | Water reservoir impoundment | Kariba | d | d | a | d | b | b | a | d | a | 0.55 |
| 160006 | Water reservoir impoundment | Kremasta | x | x | x | x | x | x | x | x | x |  |
| 160007 | Water reservoir impoundment | Hsinfengkiang (Hsingfengchiang, Xinfengjiang) | d | d | d | d | b | a | a | a | d | 0.86 |
| 160008 | Water reservoir impoundment | Killari | d | d | d | d | d | d | c | c | a | 0.91 |
| 160009 | Water reservoir impoundment | Srinagarind | d | d | d | d | c | c | c | d | a | 0.89 |
| 160010 | Water reservoir impoundment | Oroville, California | d | d | d | d | a | a | a | d | a | 0.89 |
| 160011 | Water reservoir impoundment | Marathon | d | a | a | a | a | a | a | a | a | 0.03 |
| 160012 | Water reservoir impoundment | Campotosto | d | d | a | d | b | a | a | a | a | 0.55 |
| 160013 | Water reservoir impoundment | Aswan | d | d | d | d | a | a | a | d | a | 0.89 |
| 160014 | Water reservoir impoundment | Pournari | d | d | d | d | b | b | c | d | a | 0.83 |
| 160015 | Water reservoir impoundment | Warragamba (Varragamba) | d | d | d | d | a | a | a | d | a | 0.89 |
| 160016 | Water reservoir impoundment | Asomata | d | d | a | d | c | a | a | d | a | 0.61 |
| 160017 | Water reservoir impoundment | Monteynard | d | d | a | d | c | c | a | a | a | 0.58 |
| 160018 | Water reservoir impoundment | Akosombo | x | x | x | x | x | x | x | x | x |  |
| 160019 | Water reservoir impoundment | Kinnersani | x | x | x | x | x | x | x | x | x |  |
| 160020 | Water reservoir impoundment | Charvak | x | x | x | x | x | x | x | x | x |  |
| 160021 | Water reservoir impoundment | Coyote Valley (Anderson Dam), California | x | x | x | x | x | x | x | x | x |  |
| 160022 | Water reservoir impoundment | Shenwo/Shenwu | x | x | x | x | x | x | x | x | x |  |
| 160023 | Water reservoir impoundment | Sfikia | d | d | a | d | c | c | a | d | a | 0.61 |
| 160024 | Water reservoir impoundment | Xiluodo | d | d | d | d | c | c | c | d | a | 0.89 |
| 160025 | Water reservoir impoundment | Three Gorges | d | d | d | d | b | a | c | d | a | 0.86 |
| 160026 | Water reservoir impoundment | Hoover Dam (Lake Mead), Nevada/Arizona | d | a | a | d | a | a | a | a | a | 0.30 |
| 160027 | Water reservoir impoundment | Eucumbene | x | x | x | x | x | x | x | x | x |  |
| 160028 | Water reservoir impoundment | Benmore | x | x | x | x | x | x | x | x | x |  |
| 160029 | Water reservoir impoundment | Warna (Warana) | d | d | a | d | d | a | a | a | a | 0.61 |
| 160030 | Water reservoir impoundment | Thomson | x | x | x | x | x | x | x | x | x |  |
| 160031 | Water reservoir impoundment | Kurobe | x | x | x | x | x | x | x | x | x |  |
| 160032 | Water reservoir impoundment | Bajina Basta | x | x | x | x | x | x | x | x | x |  |
| 160033 | Water reservoir impoundment | Kerr, Montana | x | x | x | x | x | x | x | x | x |  |
| 160034 | Water reservoir impoundment | Bhatsa | d | d | d | d | c | a | c | a | d | 0.89 |
| 160035 | Water reservoir impoundment | Hoa Binh | x | x | x | x | x | x | x | x | x |  |
| 160036 | Water reservoir impoundment | Pirrís Reservoir | d | d | d | d | d | a | a | a | a | 0.89 |
| 160037 | Water reservoir impoundment | Lake Baikal | x | x | x | x | x | x | x | x | x |  |
| 160038 | Water reservoir impoundment | Canelles | x | x | x | x | x | x | x | x | x |  |
| 160039 | Water reservoir impoundment | Sefia Rud | x | x | x | x | x | x | x | x | x |  |
| 160040 | Water reservoir impoundment | Danjiangkou | x | x | x | x | x | x | x | x | x |  |
| 160041 | Water reservoir impoundment | McNaughton (Mica) | x | x | x | x | x | x | x | x | x |  |
| 160042 | Water reservoir impoundment | Anderson, Idaho | x | x | x | x | x | x | x | x | x |  |
| 160043 | Water reservoir impoundment | Song Tranh 2 | d | d | a | c | c | a | c | a | a | 0.30 |
| 160044 | Water reservoir impoundment | Enguri (Inguri) | d | d | a | d | b | a | a | c | a | 0.55 |
| 160045 | Water reservoir impoundment | Kastraki | x | x | x | x | x | x | x | x | x |  |
| 160046 | Water reservoir impoundment | Nurek | d | a | a | d | a | a | a | a | a | 0.30 |
| 160047 | Water reservoir impoundment | Toktogul | x | x | x | x | x | x | x | x | x |  |
| 160048 | Water reservoir impoundment | Lake Pukaki | d | d | d | d | b | b | c | d | a | 0.83 |
| 160049 | Water reservoir impoundment | Itoiz | d | d | a | c | b | a | a | d | a | 0.30 |
| 160050 | Water reservoir impoundment | Vouglans | d | d | a | a | a | a | a | a | a | 0.30 |
| 160051 | Water reservoir impoundment | Foziling | x | x | x | x | x | x | x | x | x |  |
| 160052 | Water reservoir impoundment | Dahua | x | x | x | x | x | x | x | x | x |  |
| 160053 | Water reservoir impoundment | Cuchillo, Nuevo León | d | d | d | d | c | a | c | d | d | 0.91 |
| 160054 | Water reservoir impoundment | Pieve de Cadore | x | x | x | x | x | x | x | x | x |  |
| 160055 | Water reservoir impoundment | Piastra | x | x | x | x | x | x | x | x | x |  |
| 160056 | Water reservoir impoundment | Itezhi-Tezhi | d | d | a | c | b | a | a | a | a | 0.28 |
| 160057 | Water reservoir impoundment | Dongjing/Dongqing | d | d | d | d | c | a | a | d | a | 0.89 |
| 160058 | Water reservoir impoundment | Clark Hill, South Carolina/Georgia | x | x | x | x | x | x | x | x | x |  |
| 160059 | Water reservoir impoundment | Montedoglio | d | d | d | d | b | b | c | c | a | 0.80 |
| 160060 | Water reservoir impoundment | Karun III | d | d | d | d | b | a | a | d | a | 0.86 |
| 160061 | Water reservoir impoundment | Porto Colômbia-Volta Grande | x | x | x | x | x | x | x | x | x |  |
| 160062 | Water reservoir impoundment | Tolors | d | d | a | d | b | a | a | d | a | 0.58 |
| 160063 | Water reservoir impoundment | Komani | x | x | x | x | x | x | x | x | x |  |
| 160064 | Water reservoir impoundment | Bratsk | x | x | x | x | x | x | x | x | x |  |
| 160065 | Water reservoir impoundment | Longtan | d | d | d | d | b | a | c | d | a | 0.86 |
| 160066 | Water reservoir impoundment | Camarillas | x | x | x | x | x | x | x | x | x |  |
| 160067 | Water reservoir impoundment | Mica | x | x | x | x | x | x | x | x | x |  |
| 160068 | Water reservoir impoundment | Manicouagan 3, Quebec | d | d | d | d | c | a | c | d | a | 0.89 |
| 160069 | Water reservoir impoundment | Lake Meredith | d | d | a | a | c | a | a | b | a | 0.28 |
| 160070 | Water reservoir impoundment | Nova Ponte | x | x | x | x | x | x | x | x | x |  |
| 160071 | Water reservoir impoundment | Tous New | d | d | d | c | b | a | c | a | a | 0.55 |
| 160072 | Water reservoir impoundment | Jocassee, South Carolina | d | d | d | d | c | a | c | d | a | 0.89 |
| 160073 | Water reservoir impoundment | Yacyreta | x | x | x | x | x | x | x | x | x |  |
| 160074 | Water reservoir impoundment | Beni Haroun dam/reservoir and the Oued Athmania reservoir | d | d | d | d | a | a | c | a | d | 0.89 |
| 160075 | Water reservoir impoundment | Xiaowan | d | d | a | d | b | a | a | d | a | 0.58 |
| 160076 | Water reservoir impoundment | Keowee, South Carolina | x | x | x | x | x | x | x | x | x |  |
| 160077 | Water reservoir impoundment | Dhamni | x | x | x | x | x | x | x | x | x |  |
| 160078 | Water reservoir impoundment | Manicouagan 2, Quebec | x | x | x | x | x | x | x | x | x |  |
| 160079 | Water reservoir impoundment | Palisades, Idaho | x | x | x | x | x | x | x | x | x |  |
| 160080 | Water reservoir impoundment | Carmo do Cajuru | x | x | x | x | x | x | x | x | x |  |
| 160081 | Water reservoir impoundment | Capivara | x | x | x | x | x | x | x | x | x |  |
| 160082 | Water reservoir impoundment | LG 3, Quebec | d | d | d | d | a | a | a | a | a | 0.86 |
| 160083 | Water reservoir impoundment | Mangla | d | d | d | d | b | a | a | c | a | 0.83 |
| 160084 | Water reservoir impoundment | Shengjiaxia (Shenjia Xiashuiku) | x | x | x | x | x | x | x | x | x |  |
| 160085 | Water reservoir impoundment | Tucuruí | x | x | x | x | x | x | x | x | x |  |
| 160086 | Water reservoir impoundment | Lac de Salanfe | x | x | x | x | x | x | x | x | x |  |
| 160087 | Water reservoir impoundment | Blowering | x | x | x | x | x | x | x | x | x |  |
| 160088 | Water reservoir impoundment | Talbingo | x | x | x | x | x | x | x | x | x |  |
| 160089 | Water reservoir impoundment | Keban | x | x | x | x | x | x | x | x | x |  |
| 160090 | Water reservoir impoundment | Emosson | x | x | x | x | x | x | x | x | x |  |
| 160091 | Water reservoir impoundment | Idukki | x | x | x | x | x | x | x | x | x |  |
| 160092 | Water reservoir impoundment | Gandipet (Osman Sagar) | d | d | d | a | d | a | c | c | a | 0.61 |
| 160093 | Water reservoir impoundment | Ridracoli | d | d | d | d | c | a | a | d | a | 0.89 |
| 160094 | Water reservoir impoundment | Yantan | x | x | x | x | x | x | x | x | x |  |
| 160095 | Water reservoir impoundment | Czorsztyn Lake | d | d | d | a | b | a | d | a | a | 0.58 |
| 160096 | Water reservoir impoundment | Eguzon | x | x | x | x | x | x | x | x | x |  |
| 160097 | Water reservoir impoundment | Nagarjuna Sagar | x | x | x | x | x | x | x | x | x |  |
| 160098 | Water reservoir impoundment | Hitotsuse | x | x | x | x | x | x | x | x | x |  |
| 160099 | Water reservoir impoundment | Arimine | x | x | x | x | x | x | x | x | x |  |
| 160100 | Water reservoir impoundment | Kuzuryu | x | x | x | x | x | x | x | x | x |  |
| 160101 | Water reservoir impoundment | Midono | x | x | x | x | x | x | x | x | x |  |
| 160102 | Water reservoir impoundment | Makio | x | x | x | x | x | x | x | x | x |  |
| 160103 | Water reservoir impoundment | Miomote | x | x | x | x | x | x | x | x | x |  |
| 160104 | Water reservoir impoundment | Nagawado | x | x | x | x | x | x | x | x | x |  |
| 160105 | Water reservoir impoundment | Narugo | x | x | x | x | x | x | x | x | x |  |
| 160106 | Water reservoir impoundment | Ohkura | x | x | x | x | x | x | x | x | x |  |
| 160107 | Water reservoir impoundment | Tohri (Tori) | x | x | x | x | x | x | x | x | x |  |
| 160108 | Water reservoir impoundment | Uchikawa | x | x | x | x | x | x | x | x | x |  |
| 160109 | Water reservoir impoundment | Yuda | x | x | x | x | x | x | x | x | x |  |
| 160110 | Water reservoir impoundment | Wujiangdu | x | x | x | x | x | x | x | x | x |  |
| 160111 | Water reservoir impoundment | Lubuge | x | x | x | x | x | x | x | x | x |  |
| 160112 | Water reservoir impoundment | Balbina | d | c | a | a | a | a | a | a | a | 0.03 |
| 160113 | Water reservoir impoundment | Serre-Poncen | d | d | a | a | c | a | a | a | a | 0.30 |
| 160114 | Water reservoir impoundment | Caramany | d | d | a | a | c | a | c | a | a | 0.30 |
| 160115 | Water reservoir impoundment | Zhelin | x | x | x | x | x | x | x | x | x |  |
| 160116 | Water reservoir impoundment | Sriramsagar | x | x | x | x | x | x | x | x | x |  |
| 160117 | Water reservoir impoundment | Shuikou | x | x | x | x | x | x | x | x | x |  |
| 160118 | Water reservoir impoundment | Funil | d | d | a | a | d | a | c | a | b | 0.30 |
| 160119 | Water reservoir impoundment | Jirau | d | d | a | a | c | a | a | a | a | 0.30 |
| 160120 | Water reservoir impoundment | Katse | x | x | x | x | x | x | x | x | x |  |
| 160121 | Water reservoir impoundment | RO 2 | d | a | a | a | c | a | a | a | a | 0.03 |
| 160122 | Water reservoir impoundment | Oued Fodda | x | x | x | x | x | x | x | x | x |  |
| 160123 | Water reservoir impoundment | Shasta, California | x | x | x | x | x | x | x | x | x |  |
| 160124 | Water reservoir impoundment | Vajont | x | x | x | x | x | x | x | x | x |  |
| 160125 | Water reservoir impoundment | Mangalam | x | x | x | x | x | x | x | x | x |  |
| 160126 | Water reservoir impoundment | Contra | x | x | x | x | x | x | x | x | x |  |
| 160127 | Water reservoir impoundment | Grancarevo | x | x | x | x | x | x | x | x | x |  |
| 160128 | Water reservoir impoundment | Kamafusa | x | x | x | x | x | x | x | x | x |  |
| 160129 | Water reservoir impoundment | Qianjin | x | x | x | x | x | x | x | x | x |  |
| 160130 | Water reservoir impoundment | Capivari-Cachoeira | x | x | x | x | x | x | x | x | x |  |
| 160131 | Water reservoir impoundment | Paraibuna–Paraitinga | x | x | x | x | x | x | x | x | x |  |
| 160132 | Water reservoir impoundment | Jaguari | d | d | a | a | a | a | a | a | a | 0.30 |
| 160133 | Water reservoir impoundment | Kouris | x | x | x | x | x | x | x | x | x |  |
| 160134 | Water reservoir impoundment | Açu | x | x | x | x | x | x | x | x | x |  |
| 160135 | Water reservoir impoundment | Irapé | d | d | d | a | d | a | a | a | a | 0.61 |
| 160136 | Water reservoir impoundment | Rihand | d | d | a | d | b | a | a | d | a | 0.58 |
| 160137 | Water reservoir impoundment | Parambikulam | x | x | x | x | x | x | x | x | x |  |
| 160138 | Water reservoir impoundment | Ukai | x | x | x | x | x | x | x | x | x |  |
| 160139 | Water reservoir impoundment | Tsengwen (Zengwen) | x | x | x | x | x | x | x | x | x |  |
| 160140 | Water reservoir impoundment | Tarbela | d | a | a | d | b | a | a | a | a | 0.28 |
| 160141 | Water reservoir impoundment | Monticello (Fairfield), California | x | x | x | x | x | x | x | x | x |  |
| 160142 | Water reservoir impoundment | Tongjiezi | x | x | x | x | x | x | x | x | x |  |
| 160143 | Water reservoir impoundment | Nanchong | x | x | x | x | x | x | x | x | x |  |
| 160144 | Water reservoir impoundment | Hunanzhen | x | x | x | x | x | x | x | x | x |  |
| 160145 | Water reservoir impoundment | Vidra Lotru | x | x | x | x | x | x | x | x | x |  |
| 160146 | Water reservoir impoundment | Vidraru-Arges | x | x | x | x | x | x | x | x | x |  |
| 160147 | Water reservoir impoundment | Takase | x | x | x | x | x | x | x | x | x |  |
| 160148 | Water reservoir impoundment | Heron, New Mexico | d | d | a | d | c | a | a | d | a | 0.61 |
| 160149 | Water reservoir impoundment | Fierza | x | x | x | x | x | x | x | x | x |  |
| 160150 | Water reservoir impoundment | Itá | d | d | a | d | a | a | a | a | a | 0.58 |
| 160151 | Water reservoir impoundment | Barra Grande | d | d | a | a | a | a | a | a | a | 0.30 |
| 160152 | Water reservoir impoundment | Kadana | x | x | x | x | x | x | x | x | x |  |
| 160153 | Water reservoir impoundment | Miranda | d | d | a | a | d | a | c | d | a | 0.36 |
| 160154 | Water reservoir impoundment | Nanshui | x | x | x | x | x | x | x | x | x |  |
| 160155 | Water reservoir impoundment | Huangshi | x | x | x | x | x | x | x | x | x |  |
| 160156 | Water reservoir impoundment | Castanhão | a | d | a | d | c | a | c | a | a | 0.55 |
| 160157 | Water reservoir impoundment | Serra da Mesa | d | d | a | a | a | a | a | a | a | 0.30 |
| 160158 | Water reservoir impoundment | SM 3, Quebec | d | d | d | a | c | a | a | a | a | 0.58 |
| 160159 | Water reservoir impoundment | Sainte-Croix | x | x | x | x | x | x | x | x | x |  |
| 160160 | Water reservoir impoundment | Pertusillo | d | d | d | d | c | a | c | a | a | 0.86 |
| 160161 | Water reservoir impoundment | Cabin Creek, Colorado | x | x | x | x | x | x | x | x | x |  |
| 160162 | Water reservoir impoundment | Hendrik Verwoerd (Gariep) | x | x | x | x | x | x | x | x | x |  |
| 160163 | Water reservoir impoundment | Almendra | x | x | x | x | x | x | x | x | x |  |
| 160164 | Water reservoir impoundment | Schlegeis | x | x | x | x | x | x | x | x | x |  |
| 160165 | Water reservoir impoundment | Marimbondo | d | d | a | a | a | a | a | a | a | 0.30 |
| 160166 | Water reservoir impoundment | Emborcação | x | x | x | x | x | x | x | x | x |  |
| 160167 | Water reservoir impoundment | Sholayar | x | x | x | x | x | x | x | x | x |  |
| 160168 | Water reservoir impoundment | Sharavathi (Sharavati) | x | x | x | x | x | x | x | x | x |  |
| 160169 | Water reservoir impoundment | Ievorul Muntelui-Bicaz | x | x | x | x | x | x | x | x | x |  |
| 160170 | Water reservoir impoundment | Sobradinho | x | x | x | x | x | x | x | x | x |  |
| 160171 | Water reservoir impoundment | Machadinho | d | a | a | a | c | a | a | c | a | 0.03 |
| 160172 | Water reservoir impoundment | Campos Novos | d | a | a | d | c | a | a | c | a | 0.30 |
| 160173 | Water reservoir impoundment | Xingó | d | d | a | a | a | a | a | a | a | 0.30 |
| 160174 | Water reservoir impoundment | Toulnustouc | d | d | d | a | c | a | a | d | a | 0.61 |
| 160175 | Water reservoir impoundment | RO 3 | d | a | a | a | a | a | a | a | a | 0.03 |
| 160176 | Water reservoir impoundment | Mula | x | x | x | x | x | x | x | x | x |  |
| 160177 | Water reservoir impoundment | Itapebi | d | d | a | a | d | d | a | a | a | 0.36 |
| 160178 | Water reservoir impoundment | LG 2, Quebec | x | x | x | x | x | x | x | x | x |  |
| 160179 | Water reservoir impoundment | Toulnustouc | d | d | d | a | c | a | a | d | a | 0.61 |
| 160180 | Water reservoir impoundment | Grandval | d | a | a | a | c | a | a | a | a | 0.03 |
| 160181 | Water reservoir impoundment | Furnas | x | x | x | x | x | x | x | x | x |  |
| 160182 | Water reservoir impoundment | El Cenajo | x | x | x | x | x | x | x | x | x |  |
| 160183 | Water reservoir impoundment | Gordon River Power Development Storage | x | x | x | x | x | x | x | x | x |  |
| 160184 | Water reservoir impoundment | El Grado | x | x | x | x | x | x | x | x | x |  |
| 160185 | Water reservoir impoundment | La Cohilla | x | x | x | x | x | x | x | x | x |  |
| 160186 | Water reservoir impoundment | Rocky Reach, Washington | x | x | x | x | x | x | x | x | x |  |
| 160187 | Water reservoir impoundment | San Luis, California | x | x | x | x | x | x | x | x | x |  |
| 160188 | Water reservoir impoundment | Sanford, Michigan | x | x | x | x | x | x | x | x | x |  |
| 160189 | Water reservoir impoundment | Tehri | d | d | d | c | d | a | a | a | a | 0.61 |
